# Cell‐permeable high‐affinity tracers for G_q_ proteins provide structural insights, reveal distinct binding kinetics and identify small molecule inhibitors

**DOI:** 10.1111/bph.14960

**Published:** 2020-02-11

**Authors:** Markus Kuschak, Vigneshwaran Namasivayam, Muhammad Rafehi, Jan H. Voss, Jaspal Garg, Jonathan G. Schlegel, Aliaa Abdelrahman, Stefan Kehraus, Raphael Reher, Jim Küppers, Katharina Sylvester, Sonja Hinz, Michaela Matthey, Daniela Wenzel, Bernd K. Fleischmann, Alexander Pfeifer, Asuka Inoue, Michael Gütschow, Gabriele M. König, Christa E. Müller

**Affiliations:** ^1^ PharmaCenter Bonn, Pharmaceutical Institute, Pharmaceutical Chemistry I University of Bonn Bonn Germany; ^2^ Institute of Pharmacology and Toxicology University Hospital Bonn, University of Bonn Bonn Germany; ^3^ Institute of Pharmaceutical Biology University of Bonn Bonn Germany; ^4^ Medical Faculty, Institute of Physiology I, Life and Brain Center University of Bonn Bonn Germany; ^5^ Department of Systems Physiology, Medical Faculty Ruhr University Bochum Bochum Germany; ^6^ Graduate School of Pharmaceutical Sciences Tohoku University Sendai Miyagi Japan

## Abstract

**Background and Purpose:**

G proteins are intracellular switches that transduce and amplify extracellular signals from GPCRs. The G_q_ protein subtypes, which are coupled to PLC activation, can act as oncogenes, and their expression was reported to be up‐regulated in cancer and inflammatory diseases. G_q_ inhibition may be an efficient therapeutic strategy constituting a new level of intervention. However, diagnostic tools and therapeutic drugs for G_q_ proteins are lacking.

**Experimental Approach:**

We have now developed G_q_‐specific, cell‐permeable ^3^H‐labelled high‐affinity probes based on the macrocyclic depsipeptides FR900359 (FR) and YM‐254890 (YM). The tracers served to specifically label and quantify G_q_ proteins in their native conformation in cells and tissues with high accuracy.

**Key Results:**

FR and YM displayed low nanomolar affinity for Gα_q_, Gα_11_ and Gα_14_ expressed in CRISPR/Cas9 Gα_q_‐knockout cells, but not for Gα_15_. The two structurally very similar tracers showed strikingly different dissociation kinetics, which is predicted to result in divergent biological effects. Computational studies suggested a “dowel” effect of the pseudoirreversibly binding FR. A high‐throughput binding assay led to the discovery of novel G_q_ inhibitors, which inhibited G_q_ signalling in recombinant cells and primary murine brown adipocytes, resulting in enhanced differentiation.

**Conclusions and Implications:**

The Gq protein inhibitors YM and FR are pharmacologically different despite similar structures. The new versatile tools and powerful assays will contribute to the advancement of the rising field of G protein research.

**Abbreviations**
FR: FR900359, also known as UBO‐QIC
YM: YM‐254890
PRP: Platelet‐rich plasma
PPP: Platelet‐poor plasma
GPCR: G protein‐coupled receptor
IP_3_: Inositol 1,4,5‐trisphosphate
HA: Hemagglutinin
VSV‐G: Vesicular stomatitis virus G
DMEM: Dulbecco's Modified Eagle Medium
cpm: Counts per minute
BAT: Brown adipose tissue
IBMX: Isobutylmethylxanthine
DM: Differentiation medium
GM: Growth medium
ET‐1: Endothelin‐1
CNO: Clozapine‐*N*‐oxide
Dq: Gq‐coupled designer GPCR, a modified M3 muscarinic receptor activated by the synthetic agonist clozapine‐*N*‐oxide
MD: Molecular dynamics
TIP3P: Transferable intermolecular potential 3P
RMSD: Root mean square deviation
RMSF: Root mean square fluctuation
BIM‐dimer: BIM‐46187
BIM‐monomer: BIM‐46174
HTS: High‐throughput screening
ROS: reactive oxygen species

**What is already known**

- G_q_ proteins can act as oncogenes and their expression is up‐regulated in cancer and inflammation.
- The macrocyclic depsipeptides FR900359 (FR) and YM‐254890 (YM) are potent Gq protein inhibitors.

**What this study adds**

- Tritium‐labelled radiotracers for Gq proteins were developed leading to the discovery of novel Gq inhibitors.
- Hydrogenated FR and YM are structurally similar Gq inhibitors differing dramatically in their residence time.

**What is the clinical significance**

- The developed Gq tracers may be useful for diagnostic purposes, for example in cancer.
- A small molecule Gq inhibitor resulted in enhanced differentiation of brown adipocytes.

## 1 INTRODUCTION

Heterotrimeric G proteins are crucial switches transducing extracellular chemical signals to intracellular signalling cascades (Mahoney & Sunahara, 2016; Offermanns, 2000; Sunahara & Insel, 2016). They consist of three subunits: α (which binds GDP), β and γ. Upon activation by a GPCR, the α‐subunit releases https://www.guidetopharmacology.org/GRAC/LigandDisplayForward?ligandId=2410, binds https://www.guidetopharmacology.org/GRAC/LigandDisplayForward?ligandId=1742 instead and dissociates from the βγ subunits. There are four different G protein subfamilies, G_s_, G_i_, G_q_ and G_12/13_, that interact with different second messenger systems. Gα_q_ proteins activate PLC_β_ which leads to the release of inositol 1,4,5‐trisphosphate (IP_3_) and subsequent calcium mobilization resulting in a number of intracellular effects. Four different Gα_q_ proteins exist: http://www.guidetopharmacology.org/GRAC/FamilyDisplayForward?familyId=935, Gα_11_, Gα_14_ and Gα_15/16_ (Kamato et al., 2017; Mizuno & Itoh, 2009).

**Figure 1:**
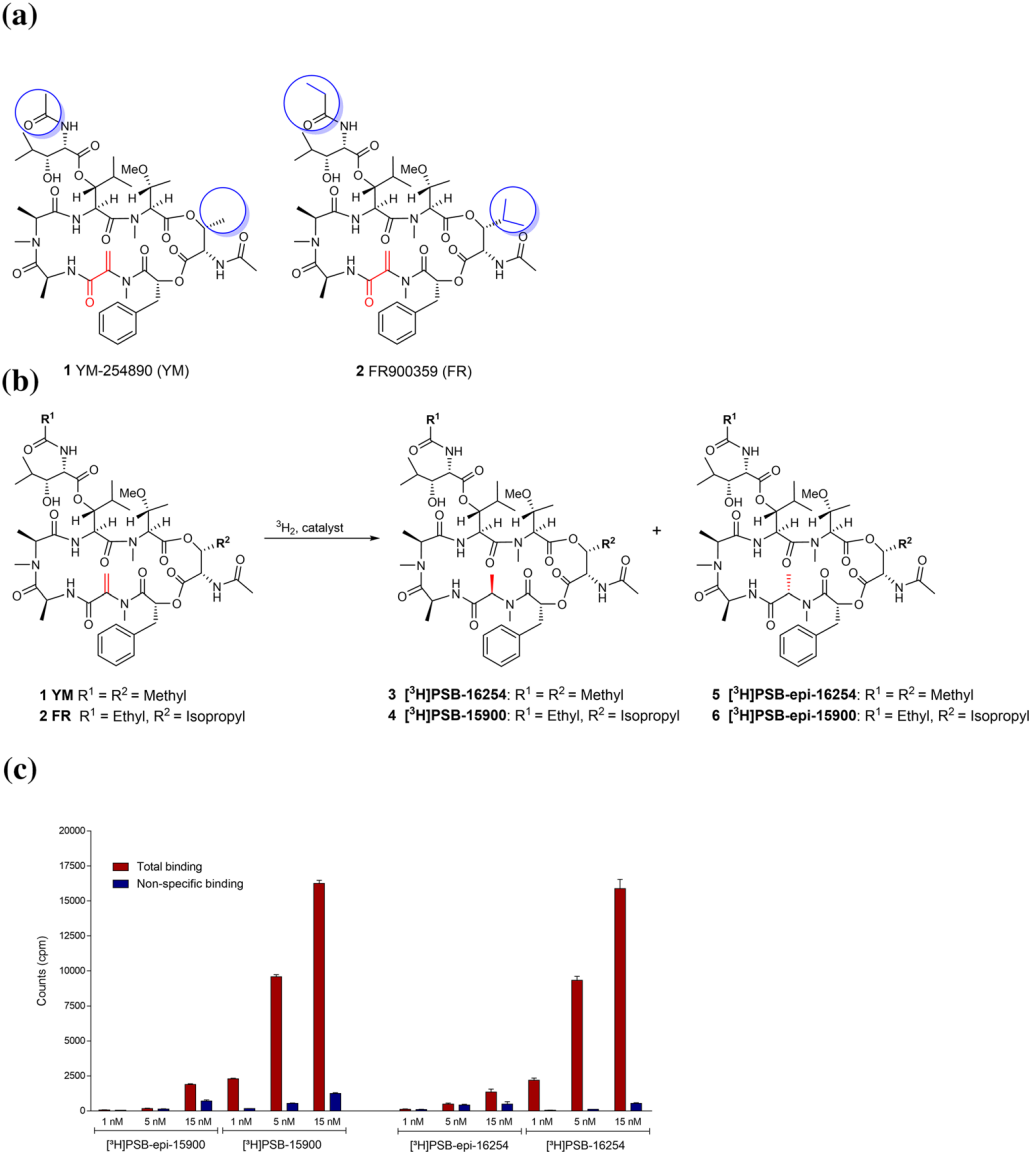
Structures, hydrogenation reaction, and preliminary binding results of G_q_ protein inhibitors. (a) Structures of the Gα_q_ protein inhibitors YM and FR. Differences between YM and FR are highlighted by blue circles; reactive partial structure is highlighted in red. (b) Labelling of YM and FR by catalytic hydrogenation with tritium gas. (c) Total binding and non‐specific binding of different concentrations of the radioligands [^3^H]PSB‐15900 (77 Ci, 2.85 TBq·mmol^−1^) and its epimer [^3^H]PSB‐epi‐15900 (32 Ci, 1.18 TBq·mmol^−1^; both FR‐derived, left) and of [^3^H]PSB‐16254 and its epimer [^3^H]PSB‐epi‐16254 (both YM‐derived, right) to human platelet membrane preparations (50 μg of protein/vial). Incubation was performed at 37°C for 1 hr. Non‐specific binding was determined in the presence of 5‐μM FR900395. Values ±SD represent data from three independent experiments

The structurally related natural macrocyclic depsipeptides, http://www.guidetopharmacology.org/GRAC/LigandDisplayForward?ligandId=9335 (**1**, Figure 1, abbreviated YM) and http://www.guidetopharmacology.org/GRAC/LigandDisplayForward?ligandId=9336 (**2**, Figure 1, abbreviated FR, also known as UBO‐QIC), have been described as potent and selective inhibitors of G_q_ proteins (Takasaki et al., 2004). YM is produced by *Chromobacterium sp.* (Taniguchi et al., 2003), while FR was isolated from the plant *Ardisia crenata* sims and is produced by the bacterial endophyte *Candidatus* Burkholderia crenata that is present as a symbiont in the leaves of the plant (Crüsemann et al., 2018; Fujioka, Koda, & Morimoto, 1988). A few analogues of FR have also been isolated, however, in tiny amounts (Crüsemann et al., 2018; Reher et al., 2018). Recently, the total syntheses of **1** and **2** and some analogues were described, but they represent labour‐intensive procedures providing only small amounts of the products; all of the synthesized analogues showed moderate potency or were inactive (Xiong et al., 2019; Zhang et al., 2017). In functional studies, FR and YM were found to be similarly potent and selective Gα_q/11_ protein inhibitors. Both are exceedingly useful for studying G_q_ protein signalling and for dissecting signalling pathways (Inamdar, Patel, Manne, Dangelmaier, & Kunapuli, 2015; Roszko et al., 2017; Schrage et al., 2015). However, more readily available inhibitors would be highly desirable. Moreover, such compounds may have potential as drugs, for example, for the treatment of chronic pulmonary disease (Matthey et al., 2017) and certain types of cancer (Feng et al., 2014).

Currently, no tools or methods are available to directly label G_q_ proteins in their native conformation, which would enable a number of biological and clinical applications. In the present study, we developed G_q_‐specific high‐affinity chemical probes for detection and quantification with high accuracy and demonstrate their power for labelling of G_q_ proteins in a variety of organs, cells and tissues. We show that the tracers derived from the structurally related G_q_ inhibitors FR and YM are in fact strikingly different possessing extremely divergent binding kinetics, which is anticipated to translate into disparity regarding their pharmacological actions. Moreover, we established a high‐throughput binding assay that has led to the discovery of small molecule G_q_ protein inhibitors, one of which—https://www.guidetopharmacology.org/GRAC/LigandDisplayForward?ligandId=10583—was further characterized and found to inhibit G_q_ signalling in recombinant cells and native brown adipocytes leading to enhanced adipocyte differentiation.

## 2 METHODS

### 2.1 Bioactive compounds

Ebselen was synthesized following reported procedures (Küppers et al., 2016; Pietka‐Ottlik, Potaczek, Piasecki, & Mlochowski, 2010). BIM‐46174 and BIM‐46178 were synthesized as described (Schmitz et al., 2014). GPCR agonists were purchased from Tocris Bioscience, Bristol, UK. FR was isolated in the laboratory of G. König as described (Schrage et al., 2015). YM was purchased from Wako Chemicals (Neuss, Germany).

### 2.2 Synthesis of radiotracers

The radioligands were prepared by Quotient Bioresearch, now Pharmaron (Cardiff, UK), by catalytic hydrogenation of YM and FR with tritium gas (custom synthesis). YM was used for preparing [^3^H]PSB‐16254 (**3**) and its epimer [^3^H]epi‐PSB‐16254 (**5**), while FR was used as a precursor for the synthesis of [^3^H]PSB‐15900 (**4**) and its epimer [^3^H]epi‐PSB‐15900 (**6**). The epimers were separated and purified by HPLC and stored as solutions of 0.2 mCi or 1 mCi·mmol^−1^ in ethanol at −20°C. The following products were obtained: [^3^H]PSB‐16254 (51 Ci, 1.89 TBq·mmol^−1^, 97.9% purity), [^3^H]epi‐PSB‐16254 (47 Ci, 1.74 TBq·mmol^−1^, 97.4% purity), [^3^H]PSB‐15900 (37 Ci, 1.37 TBq·mmol^−1^, 94.0% purity, and a second batch with 77 Ci, 2.85 TBq·mmol^−1^, 94.0% purity), and [^3^H]epi‐PSB‐15900 (32 Ci, 1.18 TBq·mmol^−1^, 79.3% purity).

### 2.3 Preparation of intact human platelet suspensions

Apheresis‐purified human thrombocyte‐plasma concentrates (PRP) were obtained from the blood bank, University of Bonn, in accordance with ethical guidelines, and directly used. For radioligand binding assays, they were diluted with 50‐mM Tris–HCl buffer, pH 7.4, to obtain a concentration of 7.5 × 10^6^ platelets/50 μl (corresponding to a 1:10 dilution).

### 2.4 Platelet membrane preparation

Apheresis‐purified human thrombocyte‐plasma concentrates were centrifuged (1,000 *g* at 4°C for 10 min), and the supernatant was decanted and centrifuged again (at 48,400 *g*, 4°C, for 60 min). The supernatant was kept and used as platelet‐poor plasma for control experiments; the formed pellet was resuspended in buffer 1 (50‐mM Tris–HCl, 5‐mM EDTA, and 150‐mM NaCl) and centrifuged at 48,400 *g*, 4°C for 60 min. The supernatant was discarded again and the pellet was resuspended in buffer 2 (5‐mM Tris–HCl and 5‐mM EDTA). The cell suspension was homogenized using an Ultra‐Turrax® (IKA Laborechnik, Staufen, Germany) for 1 min at a speed level of 4, subsequently transferred into cryovials, and stored at −80°C before use (the preparation is stable for several months). For radioligand binding assays, 50 μg of protein per vial was used, determined by the method of Bradford (Bio‐Rad Protein Assay).

### 2.5 Retroviral transfection of Gα_q_‐knockout HEK293 cells with Gα_11_, Gα_14_, Gα_15_ and Gα_q_ proteins

cDNAs encoding for the α‐subunits of the human G_11_, G_14_, G_15_ and mouse G_q_ guanine nucleotide‐binding proteins in a pcDNA3.1(+) vector were obtained from E. Kostenis. CRISPR/Cas9‐Gα_q_‐knockout (KO) HEK293 cells were from M. Yamaguchi. The protein sequences of the human and mouse Gα_q_ proteins differ only by one amino acid in position 171 (alanine in human and serine in mouse). The mouse Gα_q_ protein contained an internal hemagglutinin (HA) tag introduced through the following modifications: E125D, N126V, Y128D, V129Y and D130A. Retroviral transfection was performed as previously described (Hillmann et al., 2009). Briefly, the coding sequence of the human Gα_11_, Gα_14_, Gα_15_ and mouse Gα_q_ proteins was cloned into the pQCXIN retroviral expression vector, amplified, purified and sequenced prior to the transfection of GP^+^envAM‐12 packaging cells together with vesicular stomatitis virus G protein DNA using lipofectamine 2000. After 16 hr, 3 ml of DMEM containing 10% FBS, 1% of a penicillin/streptomycin solution (final concentrations: penicillin = 100 U·ml^−1^, streptomycin = 0.1 mg·ml^−1^), and sodium butyrate (5 mM) was added to the packaging cells. They were kept at 32°C and 5% CO_2_ for 48 hr, during which the viral vectors containing the receptor sequence were produced and released into the surrounding medium. These were harvested, filtered (45‐μm filter pore diameter), and added to HEK293 cells that had previously been modified via CRISPR/Cas9 genome editing to lack Gα_q_ expression. Polybrene solution (6 μl, 4 mg·ml^−1^ in H_2_O, filtered) was added. After 2.5 hr, the virus‐containing medium was discarded, and DMEM supplemented with 10% FBS and 1% of a penicillin/streptomycin solution (final concentrations: penicillin = 100 U·ml^−1^, streptomycin = 0.1 mg·ml^−1^) was given to the cells. These were incubated for 2 days, followed by selection of successfully transfected cells with geneticin resistance by adding G418 (200 μg·ml^−1^) to the medium. The cells were cultured in these media at 37°C and 10% CO_2_ until membrane preparations were generated.

### 2.6 Membrane preparations of recombinant HEK cells, rodent and human cancer cell lines

Recombinant HEK cells were cultured to 70% of confluence. The culture medium was discarded, and cells were washed once with approximately 5 ml of PBS. Dishes were stored at −20°C until further use. After defrosting, cells were detached with a rubber scraper after adding 5‐mM Tris–HCl, 2‐mM EDTA, pH 7.4. The collected cell suspension was homogenized using an Ultra‐Turrax® for 1 min at a speed level of 4. The cell suspension was transferred into tubes and centrifuged for 10 min at 1,000 *g*, 4°C. The pellet (P1) was discarded, and the supernatant was centrifuged for 45 min at 48,400 *g*, 4°C. The resulting supernatant was discarded, and the pellet (P2) was resuspended in 50‐mM Tris–HCl, pH 7.4, and centrifuged again for 45 min at 48,400 *g*, 4°C. This step was repeated once again, and the suspension was subsequently homogenized using an Ultra‐Turrax® for 1 min at a speed level of 4 (IKA Laborechnik), transferred into cryovials, and stored at −80°C (for up to several months). Protein determination was performed by the method of Bradford (Bio‐Rad Protein Assay). Membrane preparations of rodent and human cancer cell lines were obtained according to the same method as described for HEK cells.

### 2.7 Rat brain membrane preparations

Rat brains obtained from Pel‐Freez® (Rogers, Arkansas, USA) were defrosted in sucrose (0.32 M) and dissected to obtain cortex and striatum, respectively. Membrane preparations were prepared, P1 from striatum, P2 from cortex, according to the methods described above for HEK cells.

### 2.8 Mouse tissue membrane preparations

Female CD1 mice (Janvier Labs, Le Genest‐Saint‐Isle, France) were housed under normal light–dark cycles with ad libitum supply of chow and water. At the age of 10–12 weeks, healthy animals were killed by cervical dislocation, and the respective organs (kidney, liver, lung, heart and brain) were dissected, snap‐frozen in liquid nitrogen, and stored at −80°C until use. Each independent experiment was conducted with tissue from an individual animal. The tissue samples were weighed and subsequently homogenized in 50‐mM Tris–HCl buffer, pH 7.4, using a bead mill (TissueLyser LT, Qiagen, Venlo, NL) for 15 min 50 strokes per second, and protein determination was performed by the method of Bradford (Bio‐Rad Protein Assay). The lysate was transferred into cryovials and stored at −80°C.

### 2.9 Protein determination

Determination of proteins in solutions was carried out by the Bradford method. The stock solution (Bio‐Rad Protein Assay) was diluted 1:5 in H_2_O, at a ratio of 50:1, added to the protein solution, and incubated for 5 min at room temperature. The protein concentrations were determined photometrically at 595 nm (using a DU® 530 spectrophotometer, Beckmann‐Coulter, Krefeld, Germany). As a reference, a standard dilution of BSA in 50‐mM Tris–HCl, pH 7.4, buffer was prepared and measured, and a regression curve for the quantification of protein concentration was calculated and applied.

### 2.10 Competition binding studies

The experiments were performed in 50‐mM Tris–HCl buffer, pH 7.4. The final assay volume of 200 μl contained 5 μl of test compound in DMSO, 50 μl of [^3^H]PSB‐15900 or [^3^H]PSB‐16254, respectively, in buffer, 5 nM of final concentration, and 100 μl of human platelet membrane preparation (50 μg of protein) or intact human platelets (7.5 × 10^6^ cells/vial). Non‐specific binding was determined in the presence of 5‐μM FR. The incubation was started by the addition of membrane preparation and was performed at 37°C for 1 hr with gentle shaking. It was terminated by rapid vacuum filtration through GF/C glass‐fibre filters using a Brandel 24‐well harvester. The filters were rinsed three times with approximately 3 ml each of ice‐cold Tris–HCl buffer, 50 mM, pH 7.4 containing 0.1% Tween 20 and 0.1% BSA, to separate bound from free radioligand. The filters were punched out and transferred to scintillation vials. Luma Safe® scintillation cocktail (2.5 ml) was added, and after 6 hr of incubation, the samples were counted for 1 min each, using a liquid scintillation counter (counting efficiency 53%). Experiments in the presence of GTP were additionally performed after preincubation of the membrane preparation with GTP for 1 hr. pIC_50_ and pK_i_ values were calculated using equations for one‐site competition as implemented in GraphPad Prism 6.01 (GraphPad Inc., La Jolla, CA, USA).

### 2.11 Kinetic binding experiments

Association experiments were performed by incubation of platelet membrane preparations (50 μg of protein/vial) or intact platelets (7.5 × 10^6^ cells/vial) with [^3^H]PSB‐15900 or [^3^H]PSB‐16254, respectively, 10 nM of final concentration, at 0°C, room temperature (21°C), or 37°C, respectively, for different periods of time ranging from 1 to 420 min depending on the incubation temperature. Incubation was performed for 3 hr at 0°C, for 1.5 hr at 21°C, and for 1 hr at 37°C to reach binding equilibrium. Dissociation was subsequently initiated by the addition of FR (5 μM). All other conditions and the remaining procedure were as described above for competition experiments. The presented association half‐lives were not corrected for radioligand dissociation. For data evaluation, raw data (in cpm) from kinetic binding experiments were taken, non‐specific binding was subtracted, and data were normalized to percentage of specific binding at time 0 (= 100%), while 0 cpm corresponded to 0%. The “one phase association” equation (*Y* = *Y*_max_ * [1 − exp(−*k*_obs_ * *X*)]) implemented in GraphPad Prism was employed to determine *t*_1/2_ from *k*_obs_. For dissociation experiments, the value at time 0 was defined as 100%, and 0 cpm were defined as 0%. The “one phase exponential decay” equation (*Y* = (*Y*_0_ − NS) * exp(−*k*_off_ * *X*)+NS) implemented in GraphPad Prism was used to determine *t*_1/2_ and *k*_off_.

### 2.12 Saturation binding studies

Different concentrations of [^3^H]PSB‐15900 or [^3^H]PSB‐16254, respectively, ranging from 1 to 60 nM were incubated with platelet membrane preparations or intact platelets for 3 hr at 0°C, 1.5 hr at 21°C, or 1 hr at 37°C. All other conditions and procedures were as described above for competition experiments. Specific binding (cpm) was calculated by subtracting non‐specific binding from total binding. Total and specific binding were fitted in GraphPad Prism 6.01 (GraphPad Inc.) with the equations “One site – Total” (*Y* = (B_max_ * *X*)/(K_D_+*X*)+NS * *X*) and “One site – Specific” (*Y* = (B_max_ * *X*)/(K_D_+*X*)), respectively, while non‐specific binding was fitted as a linear function. The pK_D_ values shown in Table 1 represent the means ± SD of all individual experiments, obtained from the “One site – Specific” fit. All cpm values were converted into B_max_ values considering the specific activity of the radioligands and the amount of protein in the assay tubes.

**Table 1:** Affinities of tracers and expression levels of G_q_ family (Gα_q/11/14_) proteins determined by saturation binding expressed as pK_D_ ± SD

	[^3^H]PSB‐15900	
Tissue and temperature	pK_D_ ± SD (nM)	B_max_ ± SD (pmol·mg^−1^ of protein)
Human platelet membranes 0°C^a^^,^ b	8.04 ± 0.09	5.44 ± 0.42
Human platelet membranes 21°C^a^^,^ b ^,^ c	8.09 ± 0.12	3.86 ± 0.12
Human platelet membranes 37°C^a^^,^ b ^,^ d	8.45 ± 0.31	4.02 ± 0.65
	[^3^H]PSB‐16254:	[^3^H]PSB‐16254:
	7.96 ± 0.10	7.09 ± 3.89
Intact human platelets 37°C^a^^,^ e	8.00 ± 0.15	10600 ± 2100 binding sites/cell
Rat brain cortical membranes 0°C^a^^,^ b	8.26 ± 0.19	19.7 ± 9.6

a Values represent data from five independent experiments performed in duplicates; apparent pK_D_ values are reported.

b For curves, see Figure 2.

c For curves, see Figure S3.

d Comparison of K_D_ values determined for [^3^H]PSB‐15900 and [^3^H]PSB‐16254 revealed a statistical significance (unpaired two‐tailed *t*‐test). The affinity of [^3^H]PSB‐15900 is likely underestimated due to its extremely low dissociation kinetics.

e For curves, see Figure S4.

### 2.13 High‐throughput screening assay

Competition binding experiments were performed as described above (for incubation conditions, washing steps, and buffers) but on 96‐well plates using a Brandel 96‐well plate harvester with GF/C Unifilters (Perkin‐Elmer). After harvesting, 50 μl per cavity of Ultima Gold scintillation cocktail (Perkin‐Elmer) was added, and counting was performed as described above. FR (1 μM) was used as a positive control showing complete inhibition.

### 2.14 Calcium mobilization assays

The functional activity of YM, FR, and Ebselen as G_q_ protein inhibitors was determined by assessing their ability to antagonize ADP‐induced calcium mobilization in 1321N1 astrocytoma cells stably transfected with the human P2Y_1_ receptor, which is a G_q_‐coupled receptor. In addition, the compounds' inhibitory effects on Carbachol‐induced calcium mobilization were studied in the same cell line, which endogenously express the G_q_‐coupled muscarinic M_3_ ACh receptor. The assays were performed according to a previously described procedure (Rafehi et al., 2017) using a NOVOstar microplate reader (BMG Labtech, Offenburg, Germany).

### 2.15 Animal studies

Animal studies are reported in compliance with the ARRIVE guidelines (Kilkenny, Browne, Cuthill, Emerson, & Altman, 2010; McGrath & Lilley, 2015) and with the recommendations made by the *British Journal of Pharmacology.*

### 2.16 Analysis of brown adipocytes

Ten‐ to 12‐week‐old pregnant wild‐type C57Bl6/J mice were purchased from Charles River and housed in pathogen‐free environment under a 12‐hr light/12‐hr dark cycle having free access to standard rodent diet and water. Brown adipose tissue (BAT)‐derived mesenchymal stem cells were isolated from interscapular BAT of newborn wild‐type mice and further cultured as previously described (Klepac et al., 2016). Specifically, 170,000 cells per well were seeded in growth medium (GM; DMEM supplemented with 10% FBS and 1% penicillin–streptomycin mixture from Sigma‐Aldrich) in a six‐well plate (Day −4); 48 hr later (Day −2), GM was replaced by differentiation medium (DM), which is GM supplemented with 20‐nM insulin and 1‐nM triiodothyronine. At Day 0, cells were treated for 48 hr with BAT induction medium (DM supplemented with 0.5‐mM isobutylmethylxanthine and 1‐μM dexamethasone). For the next 5 days, cells were treated with DM that was replenished every second day. The indicated treatments were added from Day −2 to Day +7 for 9 days to the media, endothelin‐1 (ET‐1; Tocris, 10 nM), or Ebselen (50 μM). Ebselen was added 3 hr prior to ET‐1 treatment. On Day +7, differentiation of cells into mature adipocytes was analysed using Oil Red O staining of lipid droplets. Mature brown adipocytes were washed with PBS and then fixed in 4% paraformaldehyde. After washing with PBS, cells were incubated with Oil Red O (Sigma) solution (3 mg·ml^−1^ in 60% isopropanol) for 3 hr at room temperature. In addition, qPCR and Western blot analysis of adipogenic marker PPARγ and thermogenic marker UCP‐1 was performed as previously described (Klepac et al., 2016) using the following antibodies and primers, respectively.

Antibodies: UCP1 (Thermo‐Fischer, Cat#: PA9514, rabbit dilution 1:1,000), PPARγ (Cell Signaling, Cat#: 2430S, rabbit, dilution 1:500); α‐tubulin (Dianova, Cat#: DLN‐009993). Secondary mouse or rabbit fluorescent antibodies (Cell Signaling, Cat#: 5470S; 5257S; 5366S, dilution 1:15,000).
Primers: UCP1 (Fwd: 5′‐TAAGCCGGCTGAGATCTTGT‐3′,
Rev: 5′‐GGCCTCTACGACTCAGTC‐3′)
PPARγ (Fwd: 5′‐ACAAGACTACCCTTTACTGAAATTACCAT‐3′,
Rev: 5′‐TGCGAGTGGTCTTCCATCAC‐3′),
HPRT (Fwd: 5′‐GTCCCAGCGTCGTGATTAGC‐3′,
Rev: 5′‐TCATGACATCTCGAGCAAGTCTTT‐3′).

### 2.17 IP_1_ assay

Intracellular concentrations of the second messenger IP_1_ in brown preadipocytes were quantified using the HTRF‐IP_1_ kit (Cisbio Bioassays) following the manufacturer's instructions. Briefly, brown preadipocytes were transduced with lentiviruses carrying a Gq‐coupled designer GPCR, a modified M_3_ muscarinic receptor (Dq) that is activated by otherwise pharmacologically inactive clozapine‐*N*‐oxide (CNO; Armbruster, Li, Pausch, Herlitze, & Roth, 2007; Conklin et al., 2008). For ET‐1 experiments, non‐transduced preadipocytes were used. Cells were cultivated for 48 hr in GM (37°C, 5% CO_2_). For the assay, cells were trypsinized, and cell pellet was washed with PBS. Cell pellet was then resuspended in stimulation buffer containing 50‐mM LiCl and transferred to a 384‐well microtitre plate at a density of 100,000 cells per well in 7 μl of stimulation buffer. After 10 min of incubation, 3.5 μl of stimulation buffer containing inhibitors (50 μM of Ebselen, 1 μM of FR) were added for 1 hr followed by addition of 3.5 μl of stimulation buffer containing agonists (ET‐1, 10 nM; CNO, 10 μM) for 30 min; 3 μl of IP_1_‐d2 conjugate followed by 3 μl of europium cryptate‐labelled anti‐IP_1_ antibody dissolved in lysis buffer were added to the cells. After a further dark incubation of 1 hr at room temperature, time‐resolved fluorescence was measured at 620 and 665 nm with the EnSpire (Perkin Elmer) multimode plate reader.

### 2.18 Docking studies

The crystal structure of the heterotrimeric G protein (Gα_i/q_βγ) with the G_q_‐selective inhibitor YM (3AH8.pdb, resolution 2.9 Å) was obtained from the Protein Data Bank (Berman et al., 2000). The downloaded crystal structure was prepared by means of the Molecular Operating Environment (MOE 2016.08; Chemical Computing Group, Montreal, Quebec, Canada, 2014) protein structure preparation tool. The hydrogen atoms were assigned according to the Protonate‐3D module implemented in MOE 2016.08. The other G_q_‐selective inhibitor, FR, was docked into the binding site of the G_q_ protein using Autodock 4.2 (Morris et al., 2009). As an initial step, the co‐crystallized water molecules and the ligand were removed from the X‐ray structure. The atomic partial charges were added using AutoDockTools (Morris et al., 2009; Sanner, 1999). Three‐dimensional energy scoring grids of 60 × 60 × 60 points with a spacing of 0.375 Å were computed, and the grids were centred based on the co‐crystallized ligand, YM‐254890. During the docking simulations, the ligands were fully flexible while the residues of the receptor were treated as rigid. Fifty independent docking calculations using the *var*CPSO‐ls algorithm from PSO@Autodock implemented in AutoDock4.2 were performed and terminated after 500,000 evaluation steps (Namasivayam & Gunther, 2007). Parameters of the *var*CPSO‐ls algorithm, the cognitive and social coefficients c1 and c2, were set at 6.05 with 60 individual particles as swarm size. All the other parameters of the algorithm were set at their default values. A plausible binding mode of FR was selected on the basis of the lowest binding energy and based on visual inspection of the interactions.

### 2.19 Molecular dynamics simulation

The protein‐ligand complexes for molecular dynamics (MD) simulations were prepared using the Quick MD simulator module of CHARMM–GUI (Jo, Kim, Iyer, & Im, 2008; Lee et al., 2016). The complexes were solvated with transferable intermolecular potential 3P water molecules (Jorgensen, Chandrasekhar, Madura, Impey, & Klein, 1983) and neutralized by adding K^+^/Cl^−^ counter‐ions to a final concentration of 0.15 M. The MD simulations were carried out using the CHARMM36/CGenFF (3.0.1) force fields for protein and ligand atoms, respectively, and periodic boundaries (Huang et al., 2017). Ligand parameters were generated using the ParamChem service (https://cgenff.paramchem.org) implemented in CHARMM‐GUI. The prepared complexes were equilibrated using NAMD (Phillips et al., 2005) over 5 ns using the input files generated from CHARMM‐GUI. During the simulations, non‐bonded interactions were gradually switched off at 10 Å, and the long‐range electrostatic interactions were calculated using the Particle‐mesh Ewald method (Darden, Your, & Pedersen, 1993). The temperature was maintained at 303.15 K using the Langevin thermostat, and the pressure was maintained at 1 atm using a Berendsen barostat. Bond lengths involving hydrogen atoms were constrained using the M‐SHAKE algorithm. The equilibrated systems were subjected to 200 ns of unrestrained MD simulations run in triplicate for each protein‐ligand complex with the ACEMD engine (Acellera, High throughput Molecular Dynamics; Harvey, Giupponi, & Fabritiis, 2009). For every 0.4 ns, a frame was written into the trajectory file. MD trajectory analysis was performed with an in‐house script exploiting the RMSD trajectory tool implemented in VMD (Version 1.9.3; Humphrey, Dalke, & Schulten, 1996). The Cα atoms of all three subunits were taken into account in the RMSD plot, and only the Cα atoms in the Gα_q_‐subunit were considered for visualizing the fluctuation with the RMSF plot.

### 2.20 Statistical analysis and randomization

The data and statistical analysis comply with the recommendations of the *British Journal of Pharmacology* on experimental design and analysis in pharmacology. For all statistically evaluated studies, at least five separate experiments were performed. For all other experiments, three separate experiments were performed, each in duplicates, unless otherwise stated. Data were analysed with GraphPad Prism, Version 6.01 (GraphPad Inc.). Differences with *P* < .05 were considered significant. Data were expressed as mean ± SD unless otherwise stated. Shapiro–Wilk's test and *F* test were employed to determine normality and equal variance unless otherwise stated. In order to assess the difference between two groups, an unpaired parametric Student'*t* test was used when data showed normality and variance homogeneity. Unpaired parametric Welch's *t*‐test was employed when data showed no variance homogeneity. One‐way ANOVA was employed to evaluate the differences among three or more groups. Only if the ANOVA indicated a significant difference among means, Bonferroni correction was performed as a post hoc test.

Experiments were performed in a randomized manner. Blinding was not required since measurements were performed by unbiased instruments.

### 2.21 Nomenclature of targets and ligands

Key protein targets and ligands are hyperlinked to corresponding entries in http://www.guidetopharmacology.com (Harding et al., 2018), and are permanently archived in the Concise Guide to PHARMACOLOGY 2019/20 (Alexander et al., 2019).

## 3 RESULTS

### 3.1 Preparation of FR‐ and YM‐derived tracers

A prominent structural feature of both YM (**1**) and FR (**2**; Figure 1a) is an exocyclic double bond conjugated to a carbonyl group (see Figure 1a, indicated in red). The carbonyl function of this Michael acceptor structure forms a carboxamide with the next C‐terminal amino acid. The Michael reactivity of the acrylamide unit towards nucleophiles is further reduced due to the aminocarbonyl substituent at its α‐position. YM does not bind covalently to the Gα_q_ protein as shown by X‐ray crystallography (Nishimura et al., 2010); the double bond is merely involved in hydrophobic interactions. When analysing the structure of FR, we figured that the double bond could undergo catalytic hydrogenation and that this reaction might be utilized for the labelling of the compound.

In fact, hydrogenation of FR and YM was reported to lead to derivatives that were still capable of inhibiting G_q_ signalling, although with somewhat lower potency (Schrage et al., 2015; Taniguchi et al., 2004). Thus, we decided to perform catalytic hydrogenation of the double bond of FR and YM with tritium gas (Figure 1b), and the resulting mixtures of tritium‐labelled stereoisomers (**3**/**5** and **4**/**6**), *R*‐ and *S*‐configurated at the new stereocentre, were separated by HPLC coupled to radio and diode array detectors and analysed by MS (Figures S1 and S2). In both cases, one epimer (designated [^3^H]PSB‐16254 (**3**), derived from YM, and [^3^H]PSB‐15900 (**4**), derived from FR) showed high‐affinity binding, while much lower specific binding was seen with the other stereoisomer (see Figure 1c). The eutomers were assumed to be the *R*‐configurated epimers based on the study by Taniguchi et al. (2004). [^3^H]PSB‐15900 was obtained in 94.0% purity with a specific activity of 37 Ci·mmol^−1^ (1.37 TBq·mmol^−1^), a second batch showing 77 Ci·mmol^−1^ (2.85 TBq·mmol^−1^). [^3^H]PSB‐16254 displayed a specific activity of 51 Ci·mmol^−1^ (1.89 TBq·mmol^−1^) and a purity of 97.9%. In subsequent studies, we focused mainly on the FR‐derived high‐affinity radioligand [^3^H]PSB‐15900 but compared its performance to the corresponding YM‐derived [^3^H]PSB‐16254 in crucial experiments.

### 3.2 Development of binding assays

Native cell membrane preparations from rat brain cortex and human blood platelets, which were expected to express high G_q_ protein levels (Inamdar et al., 2015; Kawasaki et al., 2005; Kamato et al., 2017; Offermanns, 2000; Taniguchi et al., 2003), were used for establishing a filtration binding assay with [^3^H]PSB‐15900. Initially, we encountered severe problems due to extremely high filter binding and generally high non‐specific binding of the lipophilic macrocyclic tracer. The assay conditions could subsequently be improved by the use of thin glass fibre filters with a larger pore diameter (GF/C, 1.2‐μm pores) and by adding solubilizing agents to the washing buffer (for details, see Section 2). The same conditions also worked for the YM‐derived tracer [^3^H]PSB‐16254.

### 3.3 Saturation binding

Binding of [^3^H]PSB‐15900 to membrane preparations of human platelets was saturable, and apparent pK_D_ values determined at different temperatures were virtually identical (8.04–8.45; see Table 1). The pK_D_ value determined for [^3^H]PSB‐15900 at human platelet membranes was almost identical to that measured at rat brain cortical membranes, which can be explained by the remarkably high conservation of the Gα_q_ protein sequences in different species (Mizuno & Itoh, 2009). The YM‐derived [^3^H]PSB‐16254 behaved similarly displaying a somewhat lower pK_D_ value (7.96 vs. 8.45 at 37°C, *P* < .05). Saturation assays are depicted in Figures 2, S3, and S4. Both tracers showed a low level of non‐specific binding, but that of the less lipophilic YM‐derived [^3^H]PSB‐16254 was even lower as compared to the FR‐derived [^3^H]PSB‐15900 (cf. Figure 2a,b). These assays allowed the accurate determination of expression levels of G_q_ proteins with high accuracy (see Table 1). In human platelet membranes, an expression level of approximately 5 (3.86–7.09) pmol·mg^−1^ of total protein amount was determined. The expression level of Gα_q_ proteins in rat brain cortical membranes was determined to be about fourfold higher (B_max_ 19.7 pmol·mg^−1^ protein). Saturation assays at intact human platelets using [^3^H]PSB‐15900 resulted in a comparable pK_D_ value (8.00), and 10,600 ± 2,000 binding sites (Gα_q_ molecules) per platelet were detected (see Figure S4).

**Figure 2:**
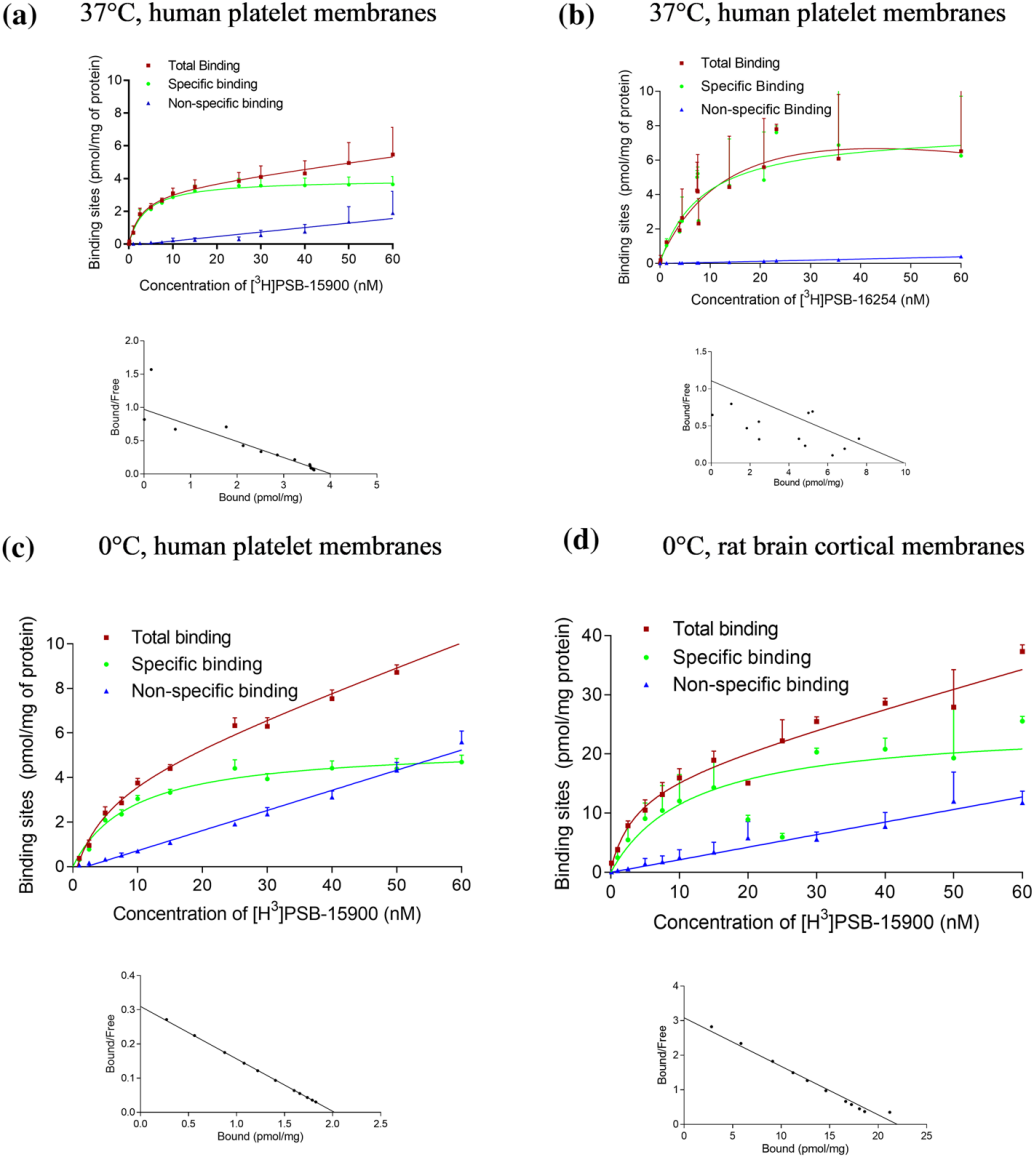
Saturation binding experiments. (a, b) Saturation binding experiments with corresponding Scatchard–Rosenthal plots of [^3^H]PSB‐15900 (a) and [^3^H]PSB‐16254 (b) at human platelet membrane preparations (50 μg of protein), incubation temperature: 37°C. The calculated apparent pK_D_ values were (a) 8.45 ± 0.31 and (b) 7.96 ± 0.10. (c, d) Saturation binding experiments and corresponding Scatchard–Rosenthal plots of [^3^H]PSB‐15900 at (c) human platelet membrane preparations (50 μg of protein) and (d) rat cortical membranes (10 μg of protein) at 0°C. The calculated apparent pK_D_ values were (c) 8.04 ± 0.09 and (b) 8.26 ± 0.19. Data are means ± SD from five independent experiments

### 3.4 Binding kinetics and computer calculations

#### 3.4.1 Kinetic studies

**Figure 3:**
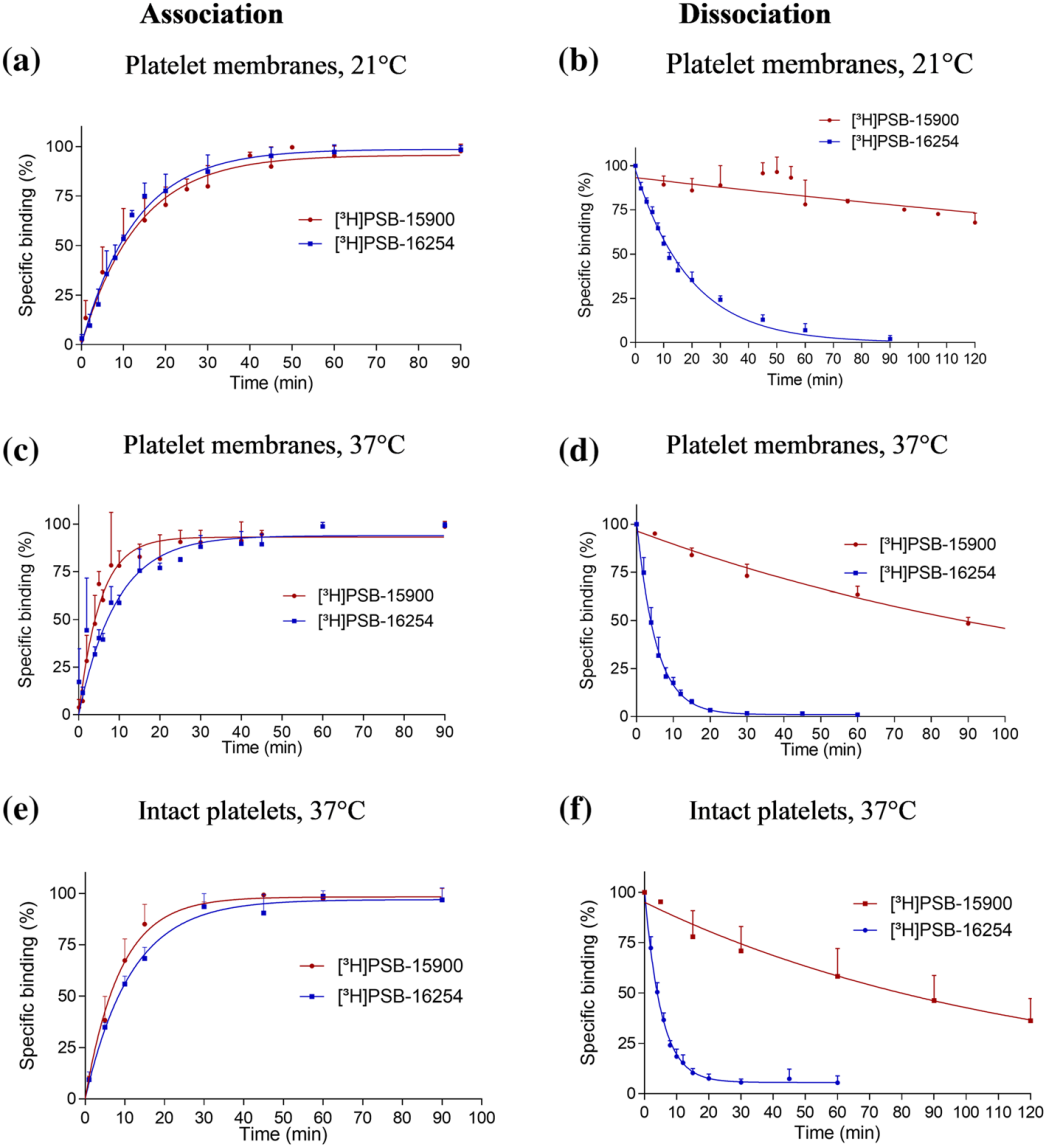
Binding kinetics. (a–d) Association and dissociation kinetics of [^3^H]PSB‐15900 and [^3^H]PSB‐16254 binding (10 nM) to membrane preparations of human platelets (50 μg of protein) performed (a, b) at 21°C and (c, d) at 37°C. The calculated *t*_1/2_ for the association of [^3^H]PSB‐15900 was 9.7 ± 4.0 min at 21°C, and 3.6 ± 1.0 min at 37°C; *t*_1/2_ for the association of [^3^H]PSB‐16254 was 8.8 ± 1.3 min at 21°C, and 6.7 ± 1.8 min at 37°C. After preincubation for 90 min with 10‐nM radioligand, dissociation was initiated by the addition of 5‐μM FR. The calculated *t*_1/2_ for the dissociation of [^3^H]PSB‐15900 was 343.3 ± 105.6 min at 21°C, and 92.1 ± 8.5 min at 37°C; *t*_1/2_ for the dissociation of [^3^H]PSB‐16254 was 13.4 ± 0.7 min at 21°C, and 3.8 ± 0.3 min at 37°C. Values represent means ± SD from five independent experiments. (e, f) Association (e) and dissociation (f) kinetics of [^3^H]PSB‐15900 and [^3^H]PSB‐16254 binding (10 nM) to intact human platelets (7.5 × 10^6^ cells/vial) performed at 37°C. Dissociation was induced after preincubation with 10‐nM radioligand for 90 min by the addition of 5‐μM FR. [^3^H]PSB‐15900 showed *t*_1/2_ values of 6.3 ± 1.5 min for the association, and of 83.5 ± 33.5 min for the dissociation. [^3^H]PSB‐16254 showed *t*_1/2_ values of 8.1 ± 0.71 min for the association, and of 3.6 ± 0.5 min for the dissociation. Values represent means ± SD from five independent experiments. The calculated kinetic pK_D_ value for [^3^H]PSB‐16254 was consonant with its saturation K_D_ value. For [^3^H]PSB‐15900, the kinetic pK_D_ value was about 10‐fold lower (9.53 ± 0.01 at 21°C, 9.37 ± 0.01 at 37°C, nM). Data points represent means ± SD from five independent experiments. All depicted association *t*_1/2_ values depict the observed association rate and were not corrected for ligand dissociation

Binding kinetics were determined for both tracers (10 nM) at different temperatures, 0°C, 21°C, and 37°C, using platelet membranes (see Figures 3, S5a, and S6a and Table S1). The FR derivative [^3^H]PSB‐15900 displayed fast, temperature‐dependent association (*t*_1/2_ = 19.7 min at 0°C, 9.7 min at 21°C, and 3.6 min at 37°C). Binding was found to be reversible; however, the dissociation rate was extremely slow (*t*_1/2_ > 450 min [>7.5 hr] at 0°C, 343.3 min at room temperature, and 92.1 min at 37°C). The interaction may therefore be described as pseudoirreversible binding. The same slow kinetics were observed at rat brain cortical membrane preparations (see Figures S5b and S6b). Surprisingly, the structurally related YM‐derived radioligand [^3^H]PSB‐16254 displayed a significantly different kinetic behaviour. While it showed only slightly slower association (*t*_1/2_ at 37°C 8.8 min for [^3^H]PSB‐16254 vs. 3.6 min for [^3^H]PSB‐15900, *P* < .05), its dissociation was rapid, much faster than that of [^3^H]PSB‐15900 (3.8 min at 37°C compared to 93.3 min for [^3^H]PSB‐16254). Kinetic values were very similar in intact human platelets as compared to membranes (see Figure 3e,f and Table S1). In contrast to [^3^H]PSB‐16254, whose kinetic pK_D_ was consonant with the saturation pK_D_ value, the kinetic K_D_ determined for [^3^H]PSB‐15900 was about 10‐fold lower than its apparent saturation K_D_ value (see Figure 3). The reason for this discrepancy may be explained by the pseudoirreversible binding of [^3^H]PSB‐15900, which would require extremely long incubation times to reach equilibrium.

#### 3.4.2 Molecular docking studies

**Figure 4:**
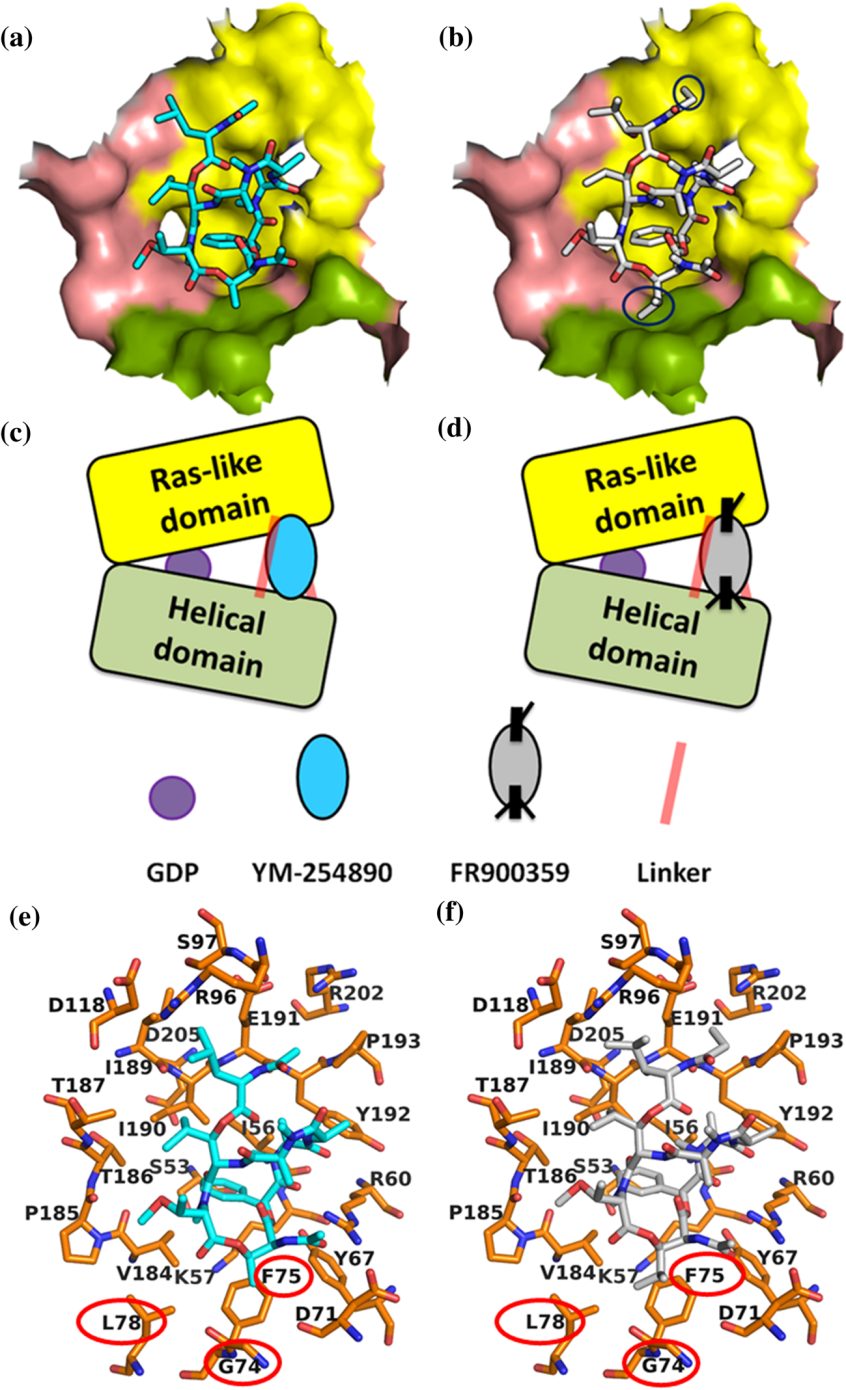
Comparison of the putative binding poses of YM and FR in the Gα_q_ protein. (a) Binding pose of YM (represented in sticks and coloured in cyan) observed in the X‐ray structure (3AH8.pdb); (b) docked pose of FR (represented in sticks and coloured in grey) in the binding pocket of the Gα_q_ protein shown in surface representation. (c, d) Schematic representation of the YM and FR binding position between the Ras‐like and the helical domain (shown in yellow and green) of the Gα_q_ protein with the two linkers connecting the domains (represented as light red‐coloured lines). The dissociation of GDP (purple coloured circle) is inhibited by YM and FR. FR (d) features additional lipophilic handles (coloured black), as compared to YM (c) which may act as latches for tighter and prolonged binding of FR between the two domains. Oxygen atoms are coloured in red, nitrogen atoms in blue, and the atoms in FR that act as latches are encircled in black in (b). (e, f) Comparison of the putative binding poses in the binding pocket of the Gα_q_ protein. (e) Binding pose for YM (coloured in cyan) observed in the X‐ray structure and (f) docked pose of FR (coloured in white) with the important amino acids (coloured in orange) in the binding pocket of the Gα_q_ protein. YM, FR, and the amino acids are represented in stick representation. Amino acids proposed to form interactions with the isopropyl group of FR and the methyl group of YM are highlighted in red. Oxygen atoms are coloured in red and nitrogen atoms in blue

YM and FR—as well as the derived tracers—are very similar molecules differing only in two residues: FR and its hydrogenation product [^3^H]PSB‐15900 contain a propionyl instead of an acetyl group (cf. R^1^ in Figure 1b) and an isopropyl instead of a methyl group (R^2^). This makes FR and its hydrogenated product [^3^H]PSB‐15900 larger and more lipophilic than YM and its derivative [^3^H]PSB‐16254. Calculated logP values (StarDrop™ software, Optibrium) are 1.37 for YM compared to 1.86 for FR. We performed docking studies to find an explanation for the very different dissociation rates of both tracers. A docking‐based model of the G_q_ protein in complex with FR was created based on the co‐crystal structure of Gαq‐βγ bound with YM (PDB ID: 3AH8, resolution: 2.9 Å; Nishimura et al., 2010). The binding poses of YM and FR were analysed, and the different substituents (R^1^ and R^2^) and their interactions within the binding pocket were compared. These docking studies allowed us to speculate that, due to its additional lipophilic “handles,” FR is anchored in the binding pocket like a dowel forming a latch, while YM lacks those anchor points and can therefore be more readily released (see Figure 4).

The docking studies also indicated that the large differences in binding affinities between the hydrogenated epimers (**3**/**5**, **4/6**, see Figure 1b) are possibly due to the formation of hydrophobic interactions between Tyr192 of the G_q_ protein and the methyl group of the *R*‐configurated stereoisomers (**3**, **4**), while in the *S*‐configurated epimers **5** and **6**, the methyl group is in a different orientation and therefore not available for hydrophobic interactions.

#### 3.4.3 Molecular dynamics simulation

To test our hypotheses, we performed MD simulations of the G_q_ protein complexes bound to either YM or FR as a next step (Figure S7a,b). The root mean square deviation (RMSD) values of the C_α_ atoms of the heterotrimeric complex rapidly reached equilibrium with approximately 1 Å deviation from the first frame of 200 ns of MD simulation (Figure S7a). The interaction pattern that emerged from the trajectory visualizations (see selected runs in Videos S1 and S2) shows that both ligands were anchored in the binding site cleft by their phenyl ring establishing a strong hydrophobic interaction with the residues Ile56, Val184, and Ile190 (for sequence alignment of G_q_ proteins and amino acid residues involved in YM and FR binding, see Figure S8). The root mean square fluctuation (RMSF) values of the Gα_q_ protein showed a similar fluctuation profile for both FR and YM (Figure S7b) indicating an analogous mechanism of interaction. An additional hydrophobic interaction was maintained between the isopropyl group of FR (R^2^ in Figure 1b) and the amino acid residues Gly74, Phe75, and Leu78 in the binding site of the Gα_q_ protein. The corresponding methyl group (R^2^) in YM formed comparably weaker interactions. The trajectory analysis also showed that the propionyl residue in FR and the corresponding acetyl group in YM (see R^1^ in Figure 1b) are likely to form weaker interactions with the Gα_q_ protein because of the high flexibility of that moiety in the binding subpocket formed by Glu191, Tyr192, Arg202, and Pro193. We assume that binding of FR and YM to the linker region of the Gα_q_ protein (also known as switch I) prevents separation of the Ras‐like domain from the helical domain (Figure 4c,d). Thus, GDP cannot easily dissociate from the Gα_q_ subunit, and the protein is arrested in its inactive state (Nishimura et al., 2010).

### 3.5 Competition binding assays

**Figure 5:**
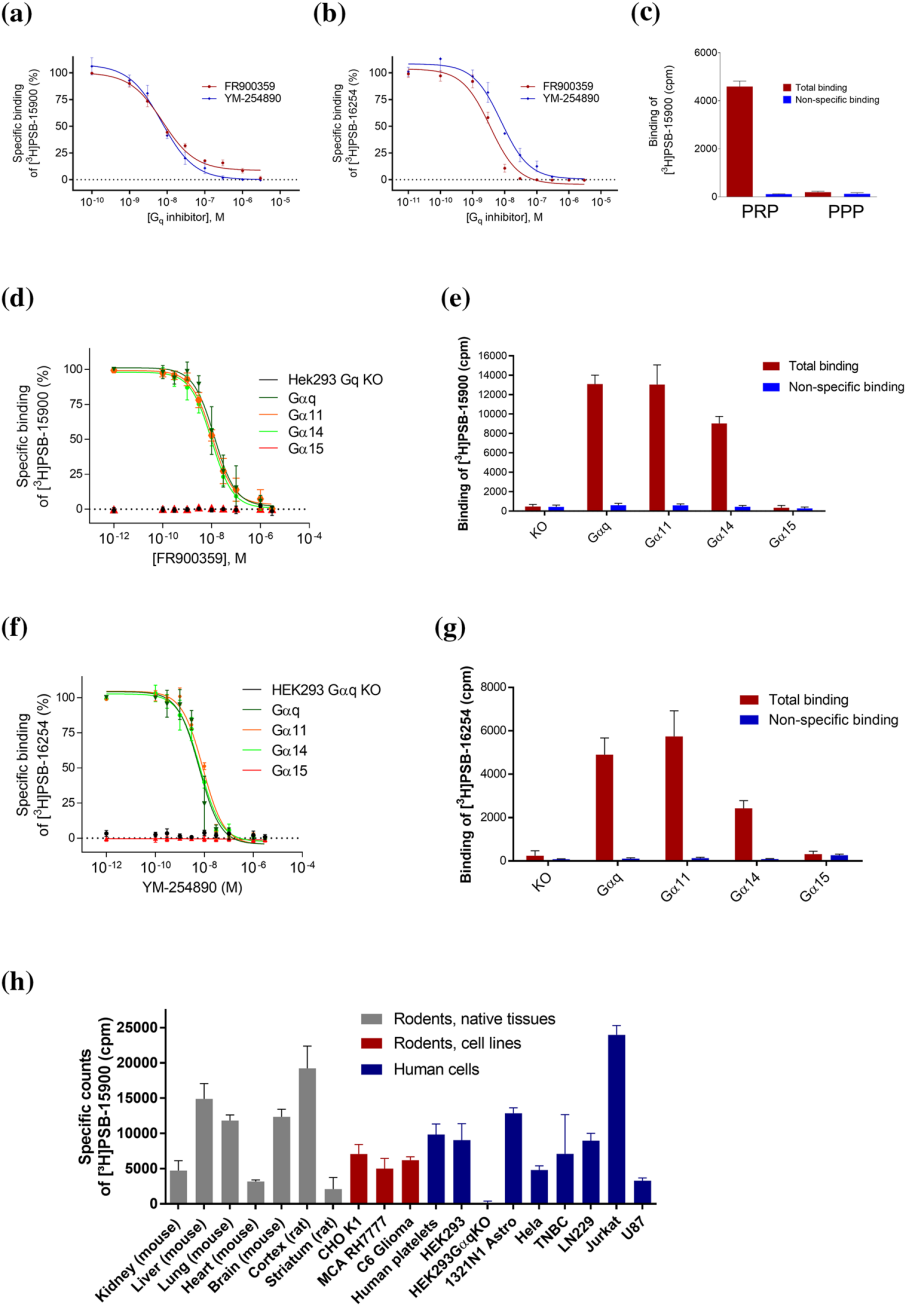
Competition binding experiments. (a, b) Competition binding experiments of FR and YM at membrane preparations of human platelets versus [^3^H]PSB‐15900 (5 nM) (a), and versus [^3^H]PSB‐16254 (5 nM) (b), performed at 37°C. Data points represent means ± SD of five independent experiments. The following pK_i_ values were calculated: 8.39 ± 0.05 (FR vs. [^3^H]PSB‐15900), 8.30 ± 0.04 (YM vs. [^3^H]PSB‐15900), 8.60 ± 0.05 (FR vs. [^3^H]PSB‐16254), and 8.20 ± 0.06 (YM vs. [^3^H]PSB‐16254). Pseudohomologous competition (FR vs. [^3^H]PSB‐15900, and YM vs. [^3^H]PSB‐16254, respectively) yielded the following K_D_ and B_max_ values: K_D_ for FR: 4.10 ± 0.50 nM, B_max_ value 6.28 ± 1.12 pmol·mg^−1^ of protein; K_D_ for YM: 6.29 ± 0.84 nM, B_max_ value 6.61 ± 0.60 pmol·mg^−1^ of protein. (c) Binding of [^3^H]PSB‐15900 (5 nM) at 37°C to PRP (7.5 million platelets/vial) and binding to platelet‐poor plasma (supernatant of PRP centrifuged at 10,000 *g* for 30 min). Values represent means ± SD of five independent experiments. (d–g) Competition binding experiments of FR versus [^3^H]PSB‐15900 (5 nM) (d, e), and YM versus [^3^H]PSB‐16254 (5 nM) (f, g) at 37°C to membrane preparations (20 μg of protein) of stably transfected HEK‐CRISPR‐Cas9‐Gα_q_‐KO cells expressing different Gα_q_ subtypes. Values represent means ± SD of five independent experiments. (h) Specific binding of [^3^H]PSB‐15900 (5 nM) to membrane preparations of tissues and cells (20 μg of total protein per vial). Non‐specific binding was determined by addition of 5‐μM FR. Values represent means ± SD of five independent experiments. Cell lines: CHO K1—CHO; MCA RH7777—rat liver hepatoma cells; C6 glioma—rat glioma cells; HEK293—HEK cells; 1321N1 astro—1321N1 astrocytoma cells (human); Hela—human cervix carcinoma cells; TNBC—human triple negative breast cancer cells; Jurkat—human Jurkat T‐lymphocytes; U87—human glioblastoma cells

Both tracers were subsequently employed for competition binding studies on human platelet membranes (at 37°C). Selected competition curves are displayed in Figure 5a,b. FR and YM showed nearly identical K_i_ values (between 3 and 5 nM) versus both radioligands, which were similar to the K_D_ values of the tracers determined in saturation binding assays indicating that all four Gα_q_ protein inhibitors, FR, YM and their hydrogenated derivatives, exhibit similar affinities. However, it should be noted that the apparent affinities for FR may be somewhat underestimated due to its extremely slow dissociation kinetics.

Since FR and its hydrogenated derivative appear to display about the same affinity and because they differ only slightly in structure, the competition of FR versus [^3^H]PSB‐15900 may be regarded as a pseudohomologous competition and consequently pseudo‐K_D_ and pseudo‐B_max_ values may be calculated yielding a pseudo‐pK_D_ value of 8.30 and a pseudo‐B_max_ value of 6.28 pmol·mg^−1^ of protein. The same calculation can be performed for YM versus its tritiated product [^3^H]PSB‐16254 resulting in a pseudo‐pK_D_ value of 8.20 and a pseudo‐B_max_ value of 6.61 pmol·mg^−1^ of protein. Pseudo‐K_D_ as well as pseudo‐B_max_ values were in agreement with those determined by the more accurate saturation binding assays (see Table 1). Pseudohomologous competition binding will be useful to simplify the procedure for estimating pK_D_ and B_max_ values in different cells and tissues.

Binding studies were also performed in living cells. PRP showed high specific binding of [^3^H]PSB‐15900, while plasma depleted from platelets did not display any specific binding (see Figure 5c). Non‐specific binding in intact platelets was low. Competition binding assays versus [^3^H]PSB‐15900 yielded virtually identical pK_i_ values of 8.72 ± 0.22 for FR and 8.72 ± 0.22 for YM (Figure S9a,b), which were well in agreement with previously determined pK_D_ values.

### 3.6 Effects of potential G_q_ protein modulators on [^3^H]PSB‐15900 binding

Next, we studied whether binding of the G_q_ tracer [^3^H]PSB‐15900 would be modulated by metal cations, nucleotides, phospholipids, agonists of G_q_ protein‐coupled receptors or the known G protein inhibitors BIM‐46187 (BIM dimer) and BIM‐46174 (BIM monomer; Ayoub et al., 2009; Schmitz et al., 2014). Effects of sodium, lithium, magnesium and calcium chloride (10 and 100 mM) on the binding of [^3^H]PSB‐15900 (5 nM) to human platelet membranes were investigated. Only Mg^2+^ (100 mM) and Ca^2+^ (10 and 100 mM) showed small effects (approximately 15–30% inhibition; Figure S10). Neither the guanine nucleotides https://www.guidetopharmacology.org/GRAC/LigandDisplayForward?ligandId=4567, GDP, and GTP (100 μM and 1 mM), nor the metabolically stable GTP analogue https://www.guidetopharmacology.org/GRAC/LigandDisplayForward?ligandId=4207 (100 μM), nor any other tested purine or pyrimidine nucleotide modulated [^3^H]PSB‐15900 binding (Figure S11). Several phospholipids, including phosphatidylcholine, ‐serine, ‐inositol, ‐ethanolamine, and phosphatidic acid at concentrations of 1 and 100 μM, also did not show any significant effect on the binding of [^3^H]PSB‐15900 (see Figure S12).

Agonists of G_q_‐coupled GPCRs are signalling by inducing a conformational change of G_q_ proteins and thus it might be possible that they affect the binding of the G_q_ inhibitor [^3^H]PSB‐15900. Therefore, we investigated agonists of G_q_‐coupled receptors that are known to be highly expressed in platelets (Koupenova & Ravid, 2018; Offermanns, 2000), including https://www.guidetopharmacology.org/GRAC/ObjectDisplayForward?objectId=347, https://www.guidetopharmacology.org/GRAC/LigandDisplayForward?ligandId=4482, https://www.guidetopharmacology.org/GRAC/ObjectDisplayForward?objectId=6 https://www.guidetopharmacology.org/GRAC/ObjectDisplayForward?objectId=20, and https://www.guidetopharmacology.org/GRAC/ObjectDisplayForward?objectId=323 receptor agonists, using membrane preparations (see Figure S13). No modulation was observed by any of the employed GPCR agonists, except for a minor effect of the PAR‐1‐selective peptide agonist https://www.guidetopharmacology.org/GRAC/LigandDisplayForward?ligandId=3742 (16% inhibition at 100 μM).

The G protein inhibitor BIM‐46187 (BIM dimer), a symmetrical disulfide, and its corresponding monomeric thiol (BIM‐46174; for structures, see Figure S14) were previously shown to directly inhibit G_q_ proteins (Schmitz et al., 2014); however, their binding site is still unknown. Binding studies on human platelet membranes versus [^3^H]PSB‐15900 (preincubation with BIM molecules for 3 hr) did not show any inhibition or modulation of radioligand binding at concentrations ranging up to 100 μM (see Figure S14).

Our results show that binding of the FR‐derived tracer [^3^H]PSB‐15900 is not allosterically modulated by compounds that are known to bind directly to G_q_ (e.g. GTP and BIM) or that affect the conformation of G_q_ proteins (e.g., GPCR agonists).

### 3.7 G_q_ subtype selectivity

Subsequently, we studied the G_q_ protein selectivity of both [^3^H]PSB‐15900 and [^3^H]PSB‐16254 by utilizing HEK (HEK293) cells whose Gα_q_ proteins had been knocked out by CRISPR/Cas9 gene editing. Using retroviral transfection (Hillmann et al., 2009), the different Gα_q_ protein subtypes, Gα_q_, Gα_11_, Gα_14_ and Gα_15_, were stably expressed in those HEK293‐G_q_‐KO cells. [^3^H]PSB‐15900 showed no specific binding to membrane preparations of HEK293‐Gα_q_‐KO cells (see Figure 5d,e), which demonstrates its selectivity for Gα_q_ proteins. In contrast, it displayed high specific binding to cell membranes recombinantly expressing Gα_q_, Gα_11_ or Gα_14_ protein, but not to those that expressed Gα_15_ (see Figure 5d,e). The same results were observed with [^3^H]PSB‐16254 (Figure 5f,g). The affinities of FR and YM for the three Gα_q_ proteins were very similar (pIC_50_ and pseudo‐B_max_ values are collected in Table S2).

### 3.8 Quantification of G_q_ proteins in different cells and tissues

In a next step, we utilized the new probes for the determination of Gα_q_ protein expression levels in different human and rodent cells, tissues, and organs (Figure 5h). Since the sequences of rat, mouse, and human Gq proteins are highly conserved, the data are directly comparable. High expression was detected in mouse liver, lung and brain, while lower levels were observed in kidney and especially in the heart. Human platelets showed a comparably high expression level. Some cancer cell lines, for example, Jurkat T cells, a human T‐cell leukaemia cell line, and human 1321N1 astrocytoma cells, were found to exhibit particularly high G_q_ expression levels (Figure 5h).

### 3.9 High‐throughput screening

**Figure 6:**
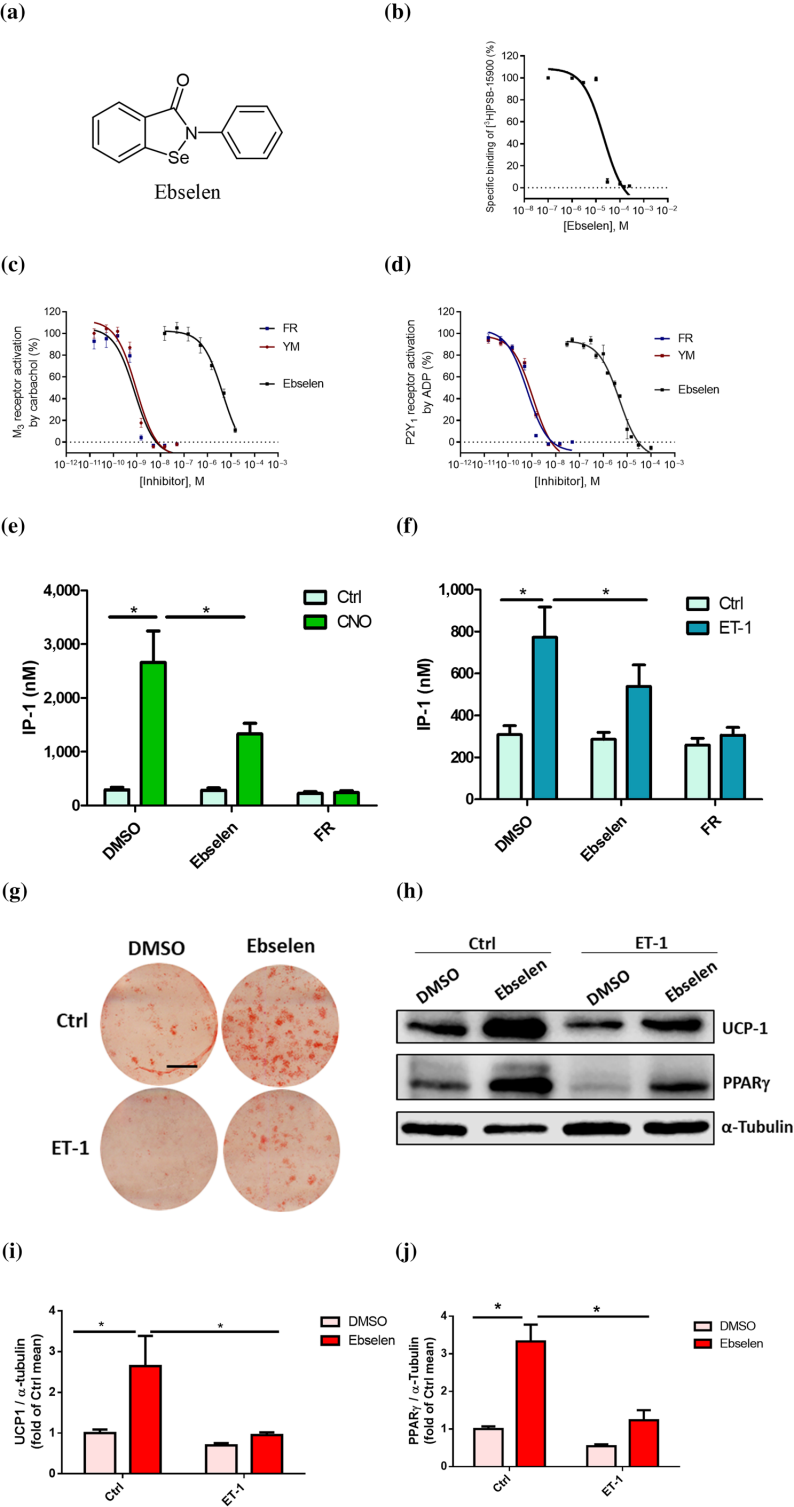
Effects of Ebselen on binding and G_q_ protein functions. (a) Structure of Ebselen; (b) concentration‐dependent inhibition of [^3^H]PSB‐15900 (5 nM) binding to human platelet membrane preparations (50 μg of protein/vial) at 37°C by Ebselen (pK_i_ value 5.03 ± 0.12). (c, d) Concentration‐dependent inhibition of calcium mobilization in 1321N1 astrocytoma cells by FR, YM, and Ebselen. Calcium mobilization was induced by activation of G_q_‐coupled receptors, (c) the endogenously expressed M_3_ receptor activated by carbachol, and (d) the recombinantly expressed P2Y_1_ receptor activated by ADP. EC_80_ concentrations of the agonists were employed. The following pIC_50_ values of the G_q_ protein inhibitors were determined: FR versus Carbachol, 9.13 ± 0.08; FR versus ADP, 9.16 ± 0.04; YM versus Carbachol, 9.05 ± 0.10; YM versus ADP, 9.02 ± 0.12; Ebselen versus Carbachol, 5.36 ± 0.16; Ebselen versus ADP, 5.63 ± 0.26 (results are means ± SD from five to six independent experiments). (e, f) IP_1_ concentrations in Gq‐DREADD expressing preadipocytes treated with Ebselen (50 μM) or FR (1 μM) in the absence (Ctrl) or presence of CNO (10 μM) (e). Data are shown as means ± SEM from 10 independent experiments. One‐way ANOVA with Bonferroni post hoc test was used, *P ≤ .05. IP_1_ levels in preadipocytes treated with Ebselen (50 μM) or FR (1 μM) in the absence (Ctrl) or presence of ET‐1 (10 nM) (f). Data are shown as means ± SEM from 10 independent experiments, one‐way ANOVA with Bonferroni post hoc test; *P ≤ .05. (g–j) Cells were treated for 9 days with indicated treatments during differentiation. Representative pictures of Oil Red O staining, scale bar 1 cm (g) and Western blot analysis (h) for thermogenic marker UCP‐1 and adipogenic marker PPARγ. α‐Tubulin was used as a loading control. Quantifications of blots (i, j) are shown as means ± SEM from five independent experiments; repeated measures of ANOVA with Bonferroni post hoc test; *P ≤ .05

Since the natural products YM and FR are complex molecules that are not easily accessible in large quantities, we established a high‐throughput screening assay for the screening of compound libraries with the aim to identify starting points for novel, more readily accessible G_q_ protein inhibitors. Thus, a binding assay with [^3^H]PSB‐15900 was established in a 96‐well format (for details, see Section 2). The assay was validated by determining the pK_i_ value of FR, which was identical to the previously determined pK_i_ value (Figure S15). A *Z*′ value of 0.69 was calculated for the assay indicating its suitability for high‐throughput screening (a value of 0.5 or greater is required; Zhang, Chung, & Oldenburg, 1999). We subsequently screened part of our in‐house compound collection, initially 2,400 compounds, which resulted in two hits (see Figure S16). One of the hit molecules, Ebselen (Noguchi, 2016; for structure, see Figure 6a), was further characterized and showed concentration‐dependent inhibition of [^3^H]PSB‐15900 binding to platelet membrane preparations with a pK_i_ value of 5.03 (Figure 6b). Ebselen was subsequently demonstrated to act as a functional inhibitor of G_q_ signalling, in a similar fashion as FR and YM. It completely blocked https://www.guidetopharmacology.org/GRAC/LigandDisplayForward?ligandId=298‐induced calcium mobilization (via the G_q_‐coupled https://www.guidetopharmacology.org/GRAC/ObjectDisplayForward?objectId=15) as well as https://www.guidetopharmacology.org/GRAC/LigandDisplayForward?ligandId=1712‐induced calcium mobilization (via the P2Y_1_ receptor) in recombinant 1321N1 astrocytoma cells with pIC_50_ values of 5.36 and 5.63, respectively (see Figure 6c,d).

### 3.10 Effects of the discovered Gq inhibitor Ebselen on brown adipocytes

Since the G_q_ signalling pathway has been described to be involved in the differentiation of BAT (Klepac et al., 2016), we used primary murine brown adipocytes to study Ebselen's G_q_ inhibitory effect. G_q_ signalling in brown adipocytes can be quantified by measuring IP_1_ production (Klepac et al., 2016). To specifically activate G_q_ signalling, we used brown adipocytes transduced with a G_q_‐coupled designer GPCR, a modified M_3_ muscarinic receptor (Dq) that is activated by otherwise pharmacologically inactive CNO (Conklin et al., 2008). Ebselen significantly reduced the CNO‐induced increase in IP_1_ levels demonstrating its ability to inhibit G_q_ signalling also in intact cells, albeit not as strongly as FR (Figure 6e). In addition, we studied the effect of Ebselen on endogenous G_q_ activators using https://www.guidetopharmacology.org/GRAC/LigandDisplayForward?ligandId=989, which we previously identified as an autocrine regulator of G_q_ signalling in brown adipocytes (Klepac et al., 2016). ET‐1 increased IP_1_ production, which was also attenuated by Ebselen pretreatment (Figure 6f). Interestingly, similar to FR (Klepac et al., 2016), Ebselen not only enhanced the differentiation of brown adipocytes but also rescued the ET‐1‐induced inhibition of brown adipogenesis as determined by Oil Red O staining of lipid droplets and expression of thermogenic (UCP‐1) and adipogenic markers (PPARγ; Figures 6g–j and S17).

## 4 DISCUSSION AND CONCLUSIONS

GPCRs constitute a prominent group of membrane proteins whose purpose is to transduce extracellular signals; they represent one of the most important classes of drug targets (Hauser, Attwood, Rask‐Andersen, Schiöth, & Gloriam, 2017; Sriram & Insel, 2018). These receptors utilize Gαβγ proteins for activating or inhibiting intracellular signalling pathways. The mechanisms by which G protein activation occurs are not well understood and currently under intensive investigation by many laboratories (Dror et al., 2015; Goricanec et al., 2016; Hilger, Masureel, & Kobilka, 2018; Smith, Lefkowitz, & Rajagopal, 2018; Venkatakrishnan et al., 2016).

### 4.1 Selective tracers and assays for Gα_q_ proteins

The structurally related macrocyclic depsipeptides YM and FR, which are produced by bacteria, were previously found to be potent, selective inhibitors of Gα_q_ proteins (Gao & Jacobson, 2016; Inamdar et al., 2015; Kamato et al., 2017;Kukkonen, 2016 ; Schrage et al., 2015). We have now prepared tritium‐labelled hydrogenated derivatives of FR and YM and developed assays to address important biological questions. Tracers with high specific activity (37–77 Ci·mmol^−1^), indicating the average introduction of 1.3–2.6 tritium atoms per molecule were obtained. The more potent stereoisomers or eutomers of both tracers (presumably *R*‐configurated) were isolated in high purity and in contrast to previous studies (Schrage et al., 2015; Taniguchi et al., 2004), they were found to be equipotent to the parent compounds YM and FR. This may be explained by a moderate purity of previously studied non‐radiolabelled hydrogenation products.

With these new tool compounds, the direct interaction of compounds with G_q_ proteins was measured in competition assays using cell membrane preparations. They provide an important complement to functional studies, which are indirect measurements that can be influenced by many factors, such as the ability to penetrate cells or various off‐target effects. The tracers bind with high affinity and selectivity to their targets, Gα_q_, Gα_11_ and Gα_14_, but not to Gα_15_, which unambiguously answers the question of the Gα_q_ subtype selectivity of FR and YM (Kukkonen, 2016; Schrage et al., 2015). This is due to the high homology of Gα_q_, Gα_11_, and Gα_14_ whose binding site for YM and FR is virtually identical, while that of the Gα_15_ protein shows lower homology (for sequence alignment, see Figure S8).

The established assays are anticipated to be highly useful for structural studies on G_q_ proteins and their complexes in order to assess and confirm the correct folding of purified G_q_ proteins used, for example, for X‐ray crystallography, NMR, or electron cryomicroscopy studies. Importantly, the tracers readily penetrate cell membranes as demonstrated for human platelets, and binding kinetics were similar in membranes as compared to intact cells (Figure 3e,f, Table S1, and Figure S9). Thus, binding studies can even be performed in living cells and comparison of competition binding of a test compound using membrane preparations versus intact cells will then provide a measure for the cell penetration of a test compound. The tracers were shown to be useful for quantitative determination of Gα_q_ expression levels in cells and tissues by saturation binding and even by straightforward and fast pseudohomologous competition binding studies with unprecedented accuracy (Table 1; Figures 2 and 5; Table S2; Figure S4). Human blood platelets, for example, were determined to express 10,600 G_q_ protein molecules per cell or approximately 4–7 pmol·mg^−1^ of protein (Table 1). In rat brain cortical membranes, the Gq expression level was about fourfold higher (20 pmol·mg^−1^ protein). This is significantly higher than the typical expression levels of GPCRs in peripheral or brain cells. We observed that some cancer cells, for example, Jurkat T cells, express particularly high Gα_q_ levels and it is known that Gα_q_ and Gα_11_ proteins can act as oncogenes (Chiariello, Marinissen, & Gutkind, 2015). Future comprehensive studies on different cancer cells and tissues, as well as immune cells, isolated from cancer patients, will be highly useful to elucidate the role of G_q_ proteins in cancer progression. Moreover, the tracers and the established assays may be used for diagnostic purposes and to test whether G_q_ proteins could serve as biomarkers for precision medicine. Tumours with high G_q_ levels may profit from treatment with G_q_ protein‐inhibiting drugs. Gα_q_ expression levels may also be altered in autoimmune diseases, such as rheumatoid arthritis (Zhang & Shi, 2016), and the expression level of Gα_q_ proteins appears to be related to inflammatory diseases, for example in the lung (John et al., 2016), to pathological conditions of the skin (Doçi et al., 2017) and even to heart diseases (Frey et al., 2017).

### 4.2 New tracers for kinetic studies

The new tracers allowed us for the first time to study the kinetics of the compounds' interaction with Gα_q_ protein. Both probes, the YM hydrogenation product [^3^H]PSB‐16254 and the FR‐derived [^3^H]PSB‐15900, displayed unexpectedly dissimilar behaviour despite their structural similarity. In contrast to the YM‐derived tracer, the FR‐derived [^3^H]PSB‐15900 showed an extraordinarily slow dissociation rate and can therefore be characterized as a pseudoirreversible binder with a *t*_1/2_ of 93.3 min at 37°C compared to only 3.5 min for [^3^H]PSB‐16254 (at 10‐nM concentration; Figure 3d). This means that YM and FR are very different and distinct pharmacological effects are to be expected; if a long residence time is required, FR would be preferred, and if fast reversibility is necessary, YM should be the method of choice for blocking G_q_ proteins. For example, for X‐ray crystallography, the long‐ and tight‐binding FR would be advantageous. Docking studies revealed a likely explanation for the observed differences. FR and its derivative contain two additional lipophilic substituents which anchor them in the binding pocket; these may function similar to a dowel thereby preventing (fast) dissociation. MD simulation studies of Gα_q_ protein in complex with FR and YM, respectively, indicated that the kinetic differences are largely contributed by the isopropyl (R^2^ in Figure 1b) interaction with Phe75 in the binding pocket. The new tracers will allow to study binding kinetics of other compounds that bind to the same site, for example, novel analogues and derivatives of YM and FR, that are accessible by chemical synthesis (Rensing, Uppal, Blumer, & Moeller, 2015; Xiong et al., 2019; Zhang et al., 2017), and by biotechnological approaches (Crüsemann et al., 2018; Taniguchi et al., 2003). The new tracers will help to design and develop G_q_ inhibitors with specific, desired kinetic profiles.

### 4.3 Tracer binding is not allosterically modulated

Interestingly, binding of [^3^H]PSB‐15900 was only slightly modulated by cations (Mg^2+^, Ca^2+^), but not by nucleotides such as GDP and GTP, or phospholipids. Moreover, agonists of G_q_ protein‐coupled receptors did not or only slightly modulate the binding of the tracer, and further G_q_ inhibitors, BIM molecules, also did not have any significant effect on the binding of the tracer. This means that the binding pocket of FR is not affected by these molecules, some of which are known to directly or indirectly interact with G_q_ proteins. This result indicates that BIM molecules do not bind to the same binding site as the Gα_q_ inhibitors FR and YM. They may either bind to a distant site as previously suggested (Schmitz et al., 2014) or require prior conjugation, for example, with GSH forming a mixed disulfide derivative, in order to interact with the FR binding site.

### 4.4 Discovery of small molecule Gα_q_ inhibitors

Because the natural products YM and FR, which are complex, large molecules, are difficult to synthesize and to produce in large enough quantities for therapeutic application, new, more easily accessible small molecule G_q_ inhibitors are desirable. To this end, we established and validated a high‐throughput competition binding assay using [^3^H]PSB‐15900. The assay was initially used for the screening of a library of 2,400 compounds consisting of proprietary synthetic compounds and natural products, approved drugs, and known bioactive molecules. This resulted in only two hits (0.08% hit rate), one of which, Ebselen, has been further characterized.

### 4.5 Ebselen blocks Gq functions in recombinant cells and native adipocytes

Our results show that the established assay is suitable for identifying novel G_q_ protein inhibitors. Ebselen is a thiol‐reactive multi‐target drug (Noguchi, 2016) that has not been previously reported to interact with heterotrimeric G proteins. It may bind to a free cysteine residue present in the Gα_q_ protein near the binding pocket of FR and thereby block the binding of the tracer. Moreover, it also inhibits G_q_ protein function as shown in calcium mobilization assays, in which G_q_ was activated via two unrelated GPCRs, the M_3_ and the P2Y_1_ receptor. Also in intact primary brown adipocytes, Ebselen decreased G_q_DREADD‐induced as well as ET‐1‐induced IP_1_ production. Like FR, Ebselen induced an increase in the differentiation of brown adipocytes.

Although Ebselen did not affect basal IP_1_ levels, it increased differentiation of brown adipocytes. This difference might be explained by the different time scales of the two experiments: IP_1_ assays were performed in washed preadipocytes over a short period of about 2 hr (acute treatment). The extracellular environment is likely devoid of any G_q_ stimulus in this setting, and, therefore, Ebselen would not affect basal IP_1_ production. However, differentiation experiments were performed over a period of 11 days (chronic treatment). Brown adipocytes are known to secrete a variety of factors during differentiation, which signal via autocrine/paracrine loops (Ali Khan et al., 2018). We previously showed that ET‐1 is one factor that is secreted by cultured brown adipocytes, which strongly inhibits differentiation in an autocrine manner via the activation of the Gq‐coupled https://www.guidetopharmacology.org/GRAC/ObjectDisplayForward?objectId=219 (Klepac et al., 2016).

Ebselen has antioxidant properties and one could speculate that this might affect adipogenic differentiation in addition to its effect on G_q_ signalling. Several studies suggested that intracellular ROS derived from https://www.guidetopharmacology.org/GRAC/FamilyDisplayForward?familyId=993, mitochondria and https://www.guidetopharmacology.org/GRAC/FamilyDisplayForward?familyId=253 increase adipocyte differentiation (Carriere, Fernandez, Rigoulet, Penicaud, & Casteilla, 2003; Furukawa et al., 2004; Kanda, Hinata, Kang, & Watanabe, 2011; Liu, Chan, Higuchi, Dusting, & Jiang, 2012; Tormos et al., 2011). Moreover, ROS production strongly increases during differentiation of preadipocytes (Furukawa et al., 2004). In accordance, oxidant molecules such as *tert*‐butylhydroperoxide and hydroxyoctadecadienoic acid (an oxidation product of linoleic acid) have been shown to increase the transcriptional activity of https://www.guidetopharmacology.org/GRAC/ObjectDisplayForward?objectId=595 (a master regulator of adipogenesis) in different cellular models (Nagy, Tontonoz, Alvarez, Chen, & Evans, 1998; Zhang, Seltmann, Zouboulis, & Konger, 2006). One would therefore anticipate an inhibition of adipogenic differentiation by the application of an antioxidant molecule. In fact, other antioxidants such as *N*‐acetyl‐l‐cysteine and Tempol blocked adipogenic differentiation in 3T3‐L1 cells (Samuni et al., 2010; Vigilanza, Aquilano, Baldelli, Rotilio, & Ciriolo, 2011). As Ebselen rather increased the adipogenic differentiation in our experiments, this effect should not be caused by its antioxidant properties but could be due to G_q_ protein inhibition.

In conclusion, we have developed probes and assays which have provided new insights and will further advance the field of GPCR and G protein research in general, and that of G_q_ proteins in particular. Future studies will be directed towards investigating the molecular mechanism of G_q_ inhibition by Ebselen and explore its suitability as a lead structure for developing potent and selective inhibitors for G_q_ proteins and eventually for other G protein subtypes.

## CONFLICT OF INTEREST

The authors declare no conflict of interest.

## AUTHOR CONTRIBUTIONS

M.K. developed and performed many of the binding assays. Some of the kinetic studies were performed, and most of the studies were analysed by J.G.S. and J.H.V. V.N. designed and supervised the molecular modelling studies, which were performed by V.N. and J.H.V. C.E.M. conceived the strategy, designed and supervised the experiments, and wrote the manuscript. A.A. developed and performed the binding assays in intact cells. S.H. devised the cloning strategy, and M.R. expressed the G_q_ proteins in HEK293‐G_q_‐KO cells and performed the calcium mobilization studies. K.S. performed binding studies in the recombinant cells and studied the effects of modulators on binding. M.K., M.R., A.A., and C.E.M. prepared the figures and tables. S.K., R.R., and G.M.K. isolated, purified, and analysed FR. J.K. synthesized BIM‐46174, BIM‐46187, and Ebselen. M.G. supervised the synthesis and analysis of the G_q_ inhibitors and had the idea of testing Ebselen. M.M., D.W., and B.F. prepared the mouse tissues. A.I. produced the HEK‐CRISPR‐Cas9‐G_q_‐KO cell line. J.G. performed the experiments in brown adipocytes and prepared the corresponding figures; these experiments were designed and supervised by A.P. All authors contributed to discussions and to the writing of the manuscript.

## DECLARATION OF TRANSPARENCY AND SCIENTIFIC RIGOUR

This Declaration acknowledges that this paper adheres to the principles for transparent reporting and scientific rigour of preclinical research as stated in the *BJP* guidelines for https://bpspubs.onlinelibrary.wiley.com/doi/full/10.1111/bph.14207, and https://bpspubs.onlinelibrary.wiley.com/doi/full/10.1111/bph.14206, and as recommended by funding agencies, publishers, and other organizations engaged with supporting research.

## Supporting information

Video S1. Supporting Information

Video S2. Supporting Information

Table S1: Comparison of association and dissociation kinetics of [³H]PSB‐15900 and [³H]PSB‐16254
Table S2: IC50 and pseudo‐Bmax values calculated from competition binding curves for FR versus [³H]PSB‐15900 (5 nM) and YM versus [³H]PSB‐16254 (5 nM) at 37°C to membrane preparations of HEK‐Gq‐KO cells stably transfected with different Gαq protein subunits
Figure S1: High performance liquid chromatogram (a) and mass spectrum (b) of the final product [^3^H]PSB‐15900 (**4**)
Figure S2: High performance liquid chromatogram (a) and mass spectrum (b) of the final product [^3^H]PSB‐16254 (**3**)
Figure S3: Saturation binding of [³H]PSB‐15900 to human platelet membrane preparations at 21°C, and corresponding Scatchard‐Rosenthal plot
Figure S4: Saturation binding of [³H]PSB‐15900 to intact human platelets at 37°C and corresponding Scatchard‐Rosenthal plot
Figure S5: Association binding kinetics of [³H]PSB‐15900 (a) to membrane preparations of human platelets (50 μg of protein/vial), and (b) to rat brain cortical membrane preparations at 0°C
Figure S6: Dissociation kinetics of [³H]PSB‐15900 10 nM (a) from membrane preparations of human platelet membranes and (b) from rat brain cortical membrane preparations at 0°C
Figure S7: Molecular dynamics simulations of Gαq protein complexes with the inhibitors FR and YM
Figure S8: Alignment of human Gαq protein subunits
Figure S9: Competition binding studies of YM (a) and FR (b) versus [³H]PSB‐15900 in intact human platelets at 37°C
Figure S10: Specific binding of 5 nM [³H]PSB‐15900 to human platelet membrane preparations in the presence of selected mono‐ and divalent metal chlorides
Figure S11: Specific binding of 5 nM [³H]PSB‐15900 to human platelet membrane preparations in the presence of nucleotides
Figure S12: Specific binding of 5 nM [³H]PSB‐15900 to human platelet membrane preparations in the presence of phospholipids
Figure S13: Specific binding of 5 nM [³H]PSB‐15900 to human platelet membrane preparations in the presence of GPCR agonists
Figure S14: Competition binding studies on intact human platelets with (A) BIM‐46174 and (B) BIM‐46187 versus [³H]PSB‐15900 (5 nM) at 37°C
Figure S15: High‐throughput screening of compound library
Figure S16: Competition binding studies of FR versus [³H]PSB‐15900 (5 nM) performed in a standard 24‐ and a high‐throughput‐96‐well format at 37°C on human platelet membrane preparations
Figure S17: Brown adipocytes were treated for 9 days with indicated treatments during the differentiation period. mRNA levels of thermogenic marker UCP‐1 (a) and adipogenic marker PPARγ (b) were determined using qPCR
