# Supplementary material for: Cell‐permeable high‐affinity tracers for Gq proteins provide structural insights, reveal distinct binding kinetics and identify small molecule inhibitors
Source: Br J Pharmacol. 2020 Feb 11;177(8):1898–916. doi: 10.1111/bph.14960 (PMC7070167; doi:10.1111/bph.14960)
Supplement: Supplementary file 3 — Table S1: Comparison of association and dissociation kinetics of [³H]PSB‐15900 and [³H]PSB‐16254 Table S2: IC50 and pseudo‐Bmax values calculated from competition binding curves for FR versus [³H]PSB‐15900 (5 nM) and YM versus [³H]PSB‐16254 (5 nM) at 37°C to membrane preparations of HEK‐Gq‐KO cells stably transfected with different Gαq protein subunits Figure S1: High performance liquid chromatogram (a) and mass spectrum (b) of the final product [3H]PSB‐15900 (4) Figure S2: High performance liquid chromatogram (a) and mass spectrum (b) of the final product [3H]PSB‐16254 (3) Figure S3: Saturation binding of [³H]PSB‐15900 to human platelet membrane preparations at 21°C, and corresponding Scatchard‐Rosenthal plot Figure S4: Saturation binding of [³H]PSB‐15900 to intact human platelets at 37°C and corresponding Scatchard‐Rosenthal plot Figure S5: Association binding kinetics of [³H]PSB‐15900 (a) to membrane preparations of human platelets (50 μg of protein/vial), and (b) to rat brain cortical membrane preparations at 0°C Figure S6: Dissociation kinetics of [³H]PSB‐15900 10 nM (a) from membrane preparations of human platelet membranes and (b) from rat brain cortical membrane preparations at 0°C Figure S7: Molecular dynamics simulations of Gαq protein complexes with the inhibitors FR and YM Figure S8: Alignment of human Gαq protein subunits Figure S9: Competition binding studies of YM (a) and FR (b) versus [³H]PSB‐15900 in intact human platelets at 37°C Figure S10: Specific binding of 5 nM [³H]PSB‐15900 to human platelet membrane preparations in the presence of selected mono‐ and divalent metal chlorides Figure S11: Specific binding of 5 nM [³H]PSB‐15900 to human platelet membrane preparations in the presence of nucleotides Figure S12: Specific binding of 5 nM [³H]PSB‐15900 to human platelet membrane preparations in the presence of phospholipids Figure S13: Specific binding of 5 nM [³H]PSB‐15900 to human platelet membrane preparations in the presence of GPCR agonists Figu [file BPH-177-1898-s003.pdf]

Supporting Information

for

Cell-permeable high-affinity tracers for G_q proteins provide structural insights, reveal distinct binding kinetics, and identify small molecule inhibitors

Markus Kuschak,[1] Vigneshwaran Namasivayam,[1] Muhammad Rafehi,[1] Jan H. Voss,[1] Jaspal Garg,[2] Jonathan G. Schlegel,[1] Aliaa Abdelrahman,[1] Stefan Kehraus,[3] Raphael Reher,[3] Jim Küppers,[1] Katharina Sylvester,[1] Sonja Hinz,[1] Michaela Matthey,[4] Daniela Wenzel,[4] Bernd Fleischmann,[4] Alexander Pfeifer,[2] Asuka Inoue,[5] Michael Gütschow,[1] Gabriele M. König,[3] Christa E. Müller[1*]

[1]PharmaCenter Bonn, Pharmaceutical Institute, Pharmaceutical Chemistry I, University of Bonn, An der Immenburg 4, D-53121 Bonn, Germany

[2]Institute of Pharmacology and Toxicology, University Hospital Bonn, University of Bonn, 53127 Bonn, Germany

[3]Institute of Pharmaceutical Biology, University of Bonn, Nussallee 6, 53113 Bonn, Germany

[4]Institute of Physiology I, Life and Brain Center, University of Bonn, Sigmund-Freud-Straße 25, 53105 Bonn, Germany

[5]Tohoku University, Graduate School of Pharmaceutical Sciences, Sendai, Miyagi, 980-8578 Japan

List of Contents

**Tables and Figures**	**Page**
**Table S1:** Comparison of association and dissociation kinetics of [^{3}H]PSB-15900 and [^{3}H]PSB-16254	**4**
**Table S2:** IC_{50} and pseudo-B_{max} values calculated from competition binding curves for FR versus [^{3}H]PSB-15900 (5 nM) and YM versus [^{3}H]PSB-16254 (5 nM) at 37°C to membrane preparations of HEK-G_q-KO cells stably transfected with different $G\alpha_q$ protein subunits	**5**
**Figure S1:** High performance liquid chromatogram (**a**) and mass spectrum (**b**) of the final product [^{3}H]PSB-15900 (**4**)	**6**
**Figure S2**: High performance liquid chromatogram (**a**) and mass spectrum (**b**) of the final product [^{3}H]PSB-16254 (**3**)	**8**
**Figure S3:** Saturation binding of [^{3}H]PSB-15900 to human platelet membrane preparations at 21°C, and corresponding Scatchard-Rosenthal plot	**10**
**Figure S4:** Saturation binding of [^{3}H]PSB-15900 to intact human platelets (at 37°C and corresponding Scatchard-Rosenthal plot	**10**
**Figure S5:** Association binding kinetics of [^{3}H]PSB-15900 (**a**) to membrane preparations of human platelets (50 µg of protein/vial), and (**b**) to rat brain cortical membrane preparations at 0°C	**11**
**Figure S6:** Dissociation kinetics of [^{3}H]PSB-15900 10 nM (**a**) from membrane preparations of human platelet membranes and (**b**) from rat brain cortical membrane preparations at 0°C	**11**
**Fig. S7:** Molecular dynamics simulations of $G\alpha_q$ protein complexes with the inhibitors FR and YM	**12**
**Fig. S8:** Alignment of human $G\alpha_q$ protein subunits	**13**
**Figure S9**: Competition binding studies of YM (**a**) and FR (**b**) versus [^{3}H]PSB-15900 in intact human platelets at 37°C	**14**
**Figure S10:** Specific binding of 5 nM [^{3}H]PSB-15900 to human platelet membrane preparations in the presence of selected mono- and divalent metal chlorides	**14**
**Figure S11:** Specific binding of 5 nM [^{3}H]PSB-15900 to human platelet membrane preparations in the presence of nucleotides	**15**
**Figure S12:** Specific binding of 5 nM [^{3}H]PSB-15900 to human platelet membrane preparations in the presence of phospholipids	**15**
**Figure S13:** Specific binding of 5 nM [^{3}H]PSB-15900 to human platelet membrane preparations in the presence of GPCR agonists.	**16**
**Figure S14:** Competition binding studies on intact human platelets with (**A**) BIM-46174 and (**B**) BIM-46187 versus [^{3}H]PSB-15900 (5 nM) at 37°C.	**17**

**Figure S15:** High-throughput screening of compound library	**18**
**Figure S16:** Competition binding studies of FR versus [^{3}H]PSB-15900 (5 nM) performed in a standard 24- and a high-throughput-96-well format at 37°C on human platelet membrane preparations	**19**
**Figure S 17:** Brown adipocytes were treated for 9 days with indicated treatments during the differentiation period. mRNA levels of thermogenic marker UCP-1 (**a**) and adipogenic marker PPARγ (**b**) were determined using qPCR	**19**

Table S1: Comparison of association and dissociation kinetics of [^{3}H]PSB-15900 and [^{3}H]PSB-16254 (10 nM of radioligand)

	[^{3}H]PSB-15900 $t_{1/2}$ ± SD(min)	[^{3}H]PSB-16254 $t_{1/2}$ ± SD (min)	Statistical comparison of values for [^{3}H]PSB-15900 and [^{3}H]PSB-16254
**0°C**			
Association<br>Human platelet membranes[a]	19.7 ± 1.7[a]	n.d.	-
Dissociation<br>Human platelet membranes[a]	>450[a]	n.d.	-
Association<br>Rat brain cortical membranes[a]	15.7 ± 0.2[a]	n.d.	-
Dissociation<br>Rat brain cortical membranes[a]	>450[a]	n.d.	-
**21°C (room temperature)**			
Association<br>Human platelet membranes[b]	9.7 ± 4.0	8.8 ± 1.3	ns
Dissociation<br>Human platelet membranes[b]	343.3 ± 105.6	13.4 ± 0.7	*
**37°C**			
Association<br>Human platelet membranes[b]	3.6 ± 1.0	6.7 ± 1.8	*
Dissociation<br>Human platelet membranes[b]	92.1 ± 8.5	3.8 ± 0.3	*
Association<br>Intact human platelets[b]	6.3 ± 1.5	8.1 ± 0.7	*
Dissociation<br>Intact human platelets[b]	83.5 ± 33.5	3.6 ± 0.5	*

[a]Values represent mean ± SD from three independent experiments.
[b]Values represent mean ± SD from five independent experiments.
*$p < 0.05$, significantly different

Table S2: IC_{50} and pseudo-B_{max} values calculated from competition binding curves for FR versus [^{3}H]PSB-15900 (5 nM) and YM versus [^{3}H]PSB-16254 (5 nM) at 37°C to membrane preparations of HEK-G_q-KO cells stably transfected with different $G\alpha_q$ protein subunits (20 µg of total protein/vial). Values represent mean ± SD from five independent experiments.

	**FR versus [^{3}H]PSB-15900**		**YM versus [^{3}H]PSB-16254**	
Gα-subunit	pIC_{50} ± SD	Pseudo-B_{max} ± SD (pmol/mg)	pIC_{50} ± SD	Pseudo-B_{max} ± SD (pmol/mg)
$G\alpha_q$	7.88 ± 0.06	6.09 ± 0.59	8.21 ± 0.07	6.04 ± 1.43
$G\alpha_{11}$	7.98 ± 0.04	6.23 ± 1.39	8.06 ± 0.05	6.23 ± 2.92
$G\alpha_{14}$	8.00 ± 0.04	4.37 ± 0.71	8.18 ± 0.05	3.18 ± 0.48
$G\alpha_{15}$	nd[a]	nd	nd	nd

[a]nd, no specific binding detectable

a

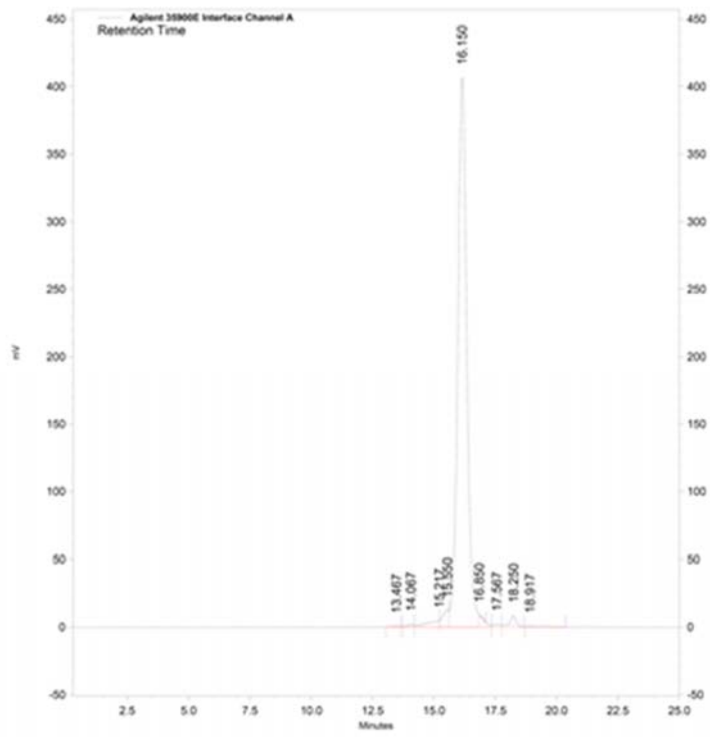
Retention Time
16.150
13.467
14.067
15.217
15.350
16.850
17.567
18.250
18.917
mV
Minutes

b

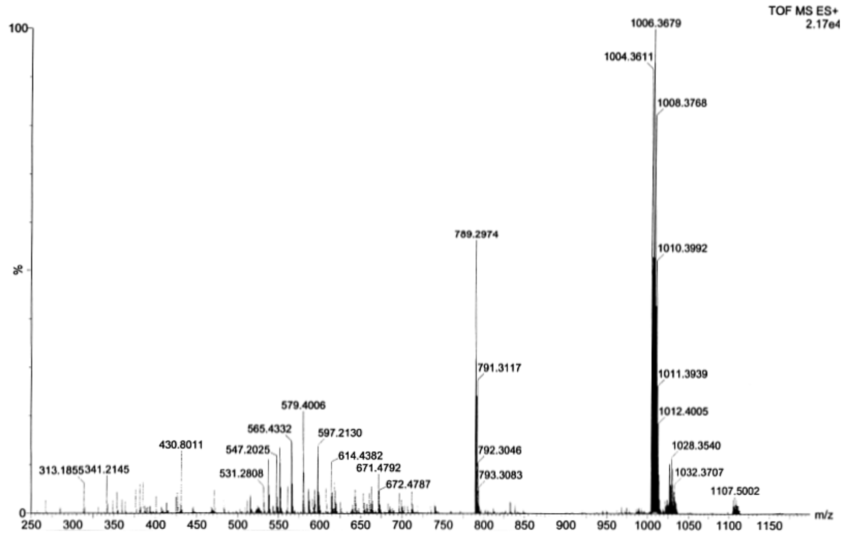
TOF MS ES+
2.17e4
1006.3679
1004.3611
1008.3768
1010.3992
1011.3939
1012.4005
1028.3540
1032.3707
1107.5002
789.2974
791.3117
792.3046
793.3083
579.4006
565.4332
597.2130
547.2025
614.4382
671.4792
672.4787
531.2808
430.8011
313.1855
341.2145
%
m/z

Figure S1: High performance liquid chromatogram (**a**) and mass spectrum (**b**) of the final product [^{3}H]PSB-15900 (**4**) on a Luna C18 column 150 x 4.6 mm, 5 µM, column temperature: 22°C. Eluent A: 2 mM ammonium acetate in water; eluent B: 2 mM ammonium acetate in methanol. Gradient: 40-100% B over 15 min, then for 10 min at 1 mL/min, injection volume: 25 µL. The HPLC apparatus was coupled to a radiodetector and a diode array detector (detection at 214 nm); molecular mass: 1006.4 g/mol; retention time: 16.15 min, purity: 94%, specific activity: 37 Ci (1.37 TBq)/mmol. A second batch was obtained with a specific activity of 77 Ci (2.85 TBq)/mmol. [^{3}H]epi-PSB-15900 (**6**) showed the same mass, a retention time of 15.37 min and a specific activity of 32 Ci (1.18 TBq)/mmol.

a

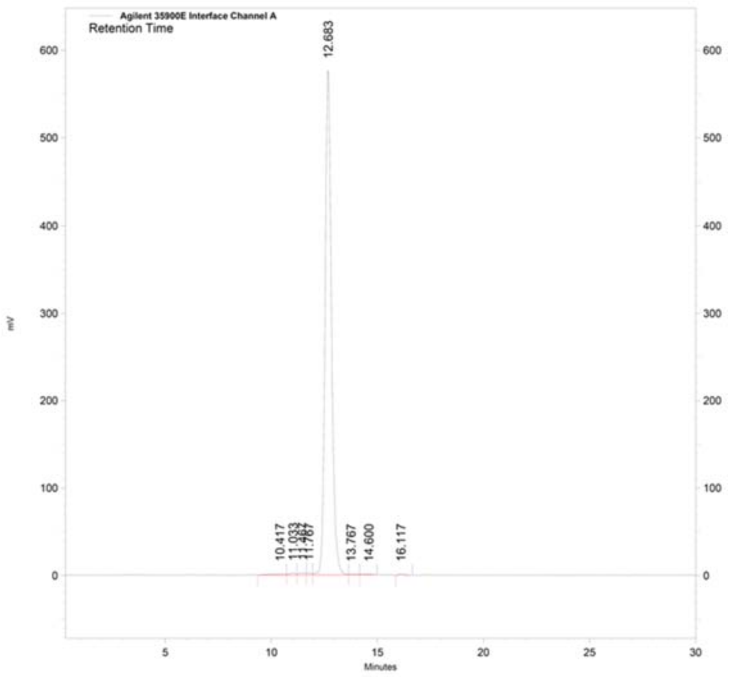

b

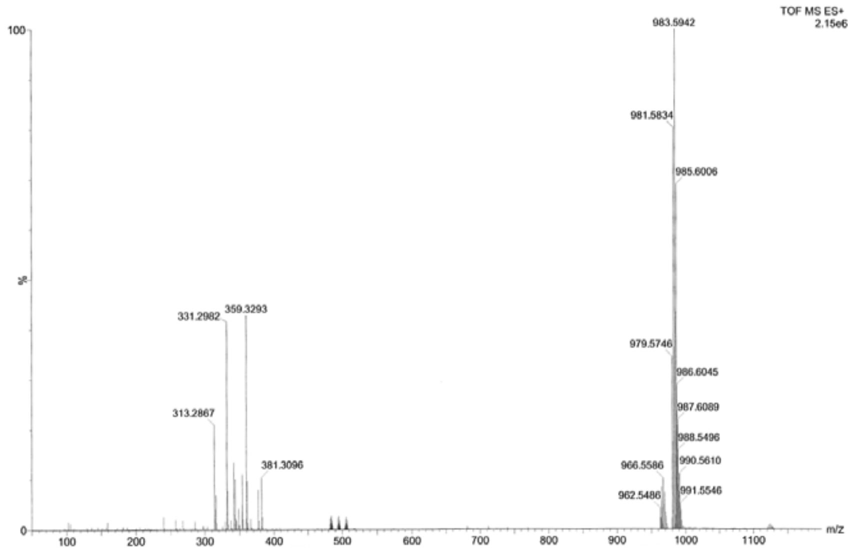

Figure S2: High performance liquid chromatogram (**a**) and mass spectrum (**b**) of the final product [^{3}H]PSB-16254 (**3**) on an Ace C18 column 5 µm, 250 x 4.6 mm, column temperature: 22°C. Eluent A: water : trifluoroacetic acid 1000:1; eluent B: methanol : trifluoroacetic acid (1000:1). Gradient:

0 min (60% B), 20 min (100% B), 25 min (100% B), 26 min (60% B), 30 min (60% B), flow rate 1.2 mL/min; injection volume: 10 µL. The HPLC apparatus was coupled to a radiodetector and a diode array detector (detection at 200 nm); molecular mass: 983.6 g/mol; retention time: 12.68 min, purity: 97.9%, specific activity: 51 Ci (1.89 TBq)/mmol. [^{3}H]epi-PSB-16254 (**5**) showed the same mass, a purity of 97.4%, a retention time of 12.73 min and a specific activity of 47 Ci (1.74 TBq)/mmol.

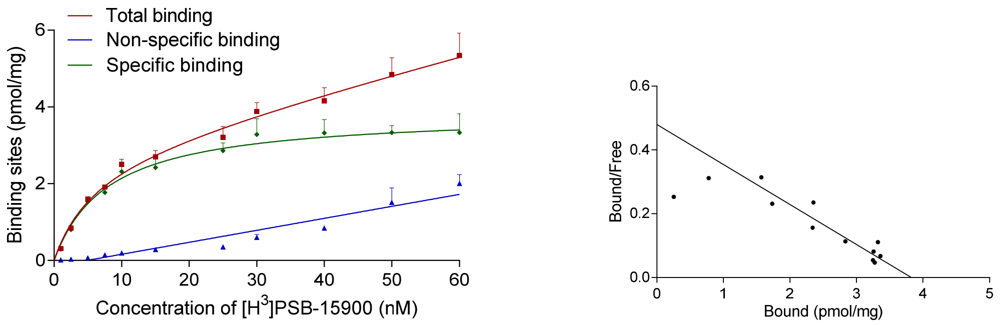

Figure S3: Saturation binding of $[^3H]$PSB-15900 to human platelet membrane preparations (50 µg of protein/vial) at 21°C, and corresponding Scatchard-Rosenthal plot. Data are means ± SD from five independent experiments. The calculated pK_D value was 8.09 ± 0.12 (K_D = 8.01 ± 2.03 nM).

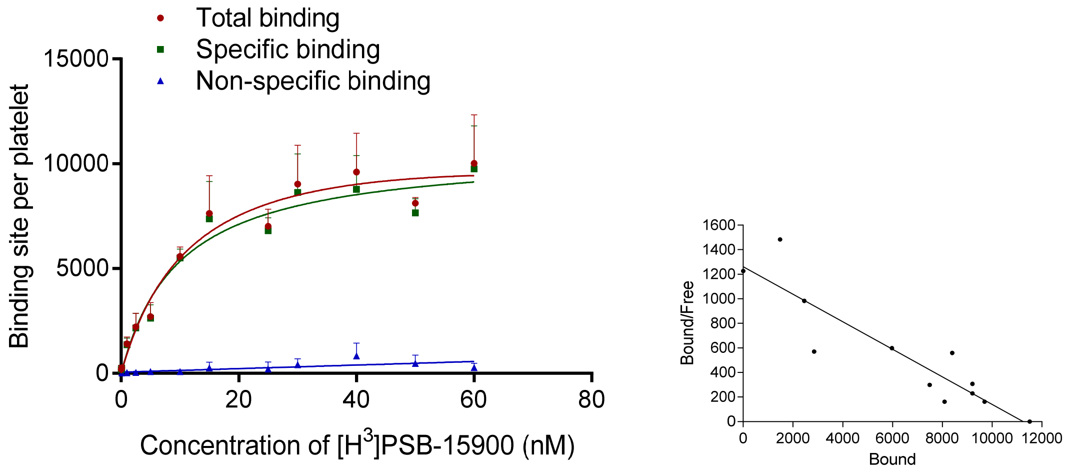

Figure S4: Saturation binding of $[^3H]$PSB-15900 to intact human platelets (7.5 x 10^6/vial) at 37°C and corresponding Scatchard-Rosenthal plot. Data are means ± SD from five independent experiments. The calculated pK_D value was 8.00 ± 0.15 (K_i = 9.92 ± 4.20 nM).

a

b

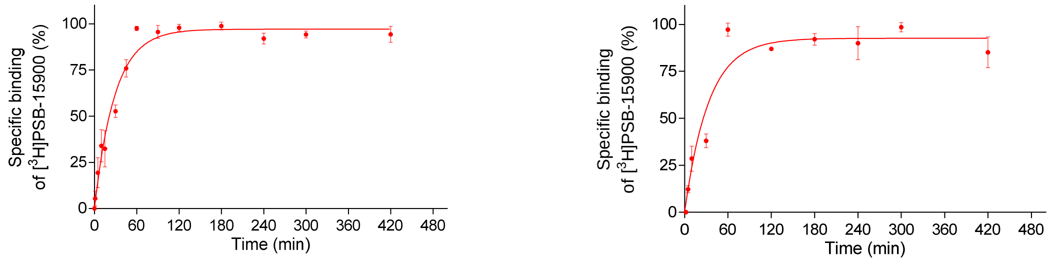

Figure S5: Association binding kinetics of [^{3}H]PSB-15900 (10 nM) (**a**) to membrane preparations of human platelets (50 µg of protein/vial), and (**b**) to rat brain cortical membrane preparations (10 µg of protein/vial) at 0°C. The calculated $t_{1/2}$ values were for (**a**) 19.7 ± 1.7 min, and for (**b**) 15.7 ± 0.2 min. Values represent means ± SD from three independent experiments.

a

b

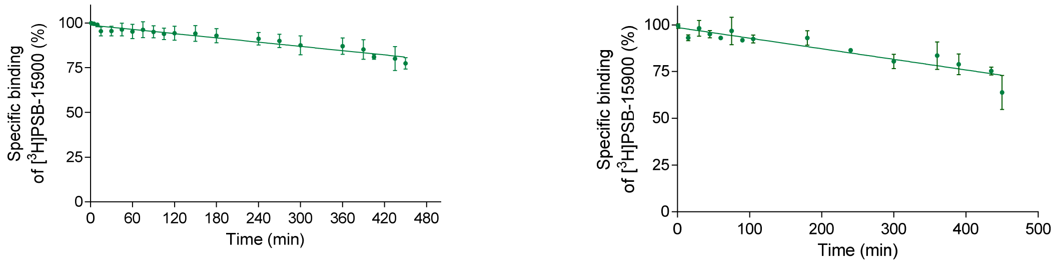

Figure S6: Dissociation kinetics of [^{3}H]PSB-15900 10 nM (**a**) from membrane preparations of human platelet membranes (50 µg of protein/vial) and (**b**) from rat brain cortical membrane preparations (10 µg of protein/vial) at 0°C. Dissociation was initiated by the addition of 5 µM of FR after preincubation with [^{3}H]PSB-15900 for 90 min. The calculated $t_{1/2}$ was >7.5 h. Values represent means ± SD from three independent experiments.

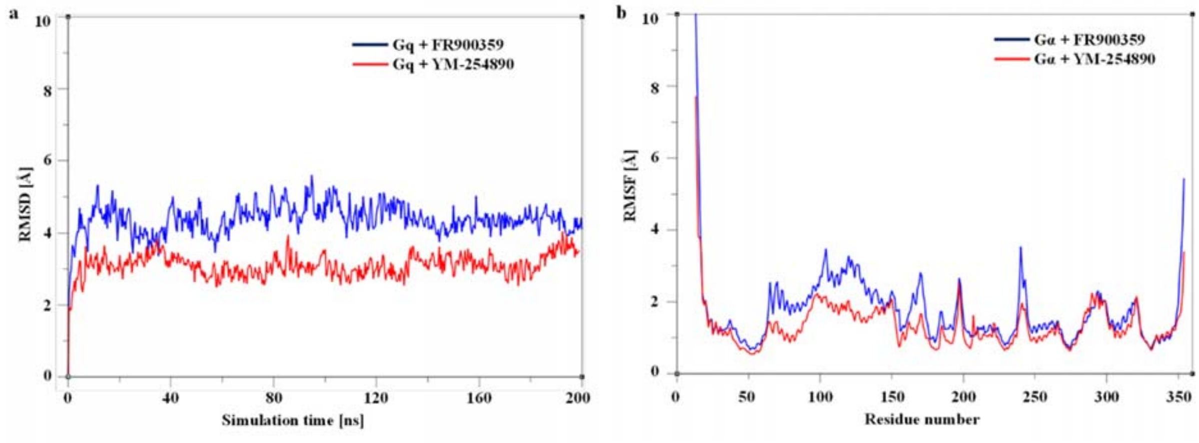

Fig. S7a,b: Molecular dynamics simulations of Gα_q protein complexes with the inhibitors FR and YM. (**a**) Root mean square deviation (RMSD) values obtained from the Cα atoms of the protein relative to the initial frame of the simulation (corresponding to the crystal structure conformation of the protein)[18], and (**b**) root mean square fluctuation (RMSF).

```
SP|P50148|GNAQ_HUMAN  MTLESI---MACCLSEEAKEARRINDEIERQLRRDKRDARRELKLLLLGTGESGKSTFIK 57
SP|P29992|GNA11_HUMAN MTLESM---MACCLSDEVKESKRINAEIEKQLRRDKRDARRELKLLLLGTGESGKSTFIK 57
SP|O95837|GNA14_HUMAN ----MA---GCCCLSAEEKESQRISAEIERQLRRDKKDARRELKLLLLGTGESGKSTFIK 53
SP|P30679|GNA15_HUMAN MARSLTWRCCPWCLTEDEKAAARVDQEINRILLEQKKQDRGELKLLLLGPGESGKSTFIK 60
                                  **: : * : *:. **:: * .:*:: * ******** **********

SP|P50148|GNAQ_HUMAN  QMRIIHGSGYSDEDKRGFTKLVYQNIFTAMQAMIRAMDTLKIPYKYEHNKAHAQLVREVD 117
SP|P29992|GNA11_HUMAN QMRIIHGAGYSEEDKRGFTKLVYQNIFTAMQAMIRAMETLKILYKYEQNKANALLIREVD 117
SP|O95837|GNA14_HUMAN QMRIIHGSGYSDEDRKGFTKLVYQNIFTAMQAMIRAMDTLRIQYVCEQNKENAQIIREVE 113
SP|P30679|GNA15_HUMAN QMRIIHGAGYSEEERKGFRPLVYQNIFVSMRAMIEAMERLQIPFSRPESKHHASLVMSQD 120
                      *******:***:*:::**  *******.:*:***.**: *:* :   ..* :* :: . :

SP|P50148|GNAQ_HUMAN  VEKVSAFENPYVDAIKSLWNDPGIQECYDRRREYQLSDSTKYYLNDLDRVADPAYLPTQQ 177
SP|P29992|GNA11_HUMAN VEKVTTFEHQYVSAIKTLWEDPGIQECYDRRREYQLSDSAKYYLTDVDRIATLGYLPTQQ 177
SP|O95837|GNA14_HUMAN VDKVSMLSREQVEAIKQLWQDPGIQECYDRRREYQLSDSAKYYLTDIDRIATPSFVPTQQ 173
SP|P30679|GNA15_HUMAN PYKVTTFEKRYAAAMQWLWRDAGIRAYYERRREFHLLDSAVYYLSHLERITEEGYVPTAQ 180
                        **: :..  . *:: **.* **:  *:****::* **: ***..::*::  .::** *

SP|P50148|GNAQ_HUMAN  DVLRVRVPTTGIIEYPFDLQSVIFRMVDVGGQRSERRKWIHCFENVTSIMFLVALSEYDQ 237
SP|P29992|GNA11_HUMAN DVLRVRVPTTGIIEYPFDLENIIFRMVDVGGQRSERRKWIHCFENVTSIMFLVALSEYDQ 237
SP|O95837|GNA14_HUMAN DVLRVRVPTTGIIEYPFDLENIIFRMVDVGGQRSERRKWIHCFESVTSIIFLVALSEYDQ 233
SP|P30679|GNA15_HUMAN DVLRSRMPTTGINEYCFSVQKTNLRIVDVGGQKSERKKWIHCFENVIALIYLASLSEYDQ 240
                      **** *:***** ** *.::.  :*:******:***:*******.* ::::*.:******

SP|P50148|GNAQ_HUMAN  VLVESDNENRMEESKALFRTIITYPWFQNSSVILFLNKKDLLEEKIMYSHLVDYFPEYDG 297
SP|P29992|GNA11_HUMAN VLVESDNENRMEESKALFRTIITYPWFQNSSVILFLNKKDLLEDKILYSHLVDYFPEFDG 297
SP|O95837|GNA14_HUMAN VLAECDNENRMEESKALFKTIITYPWFLNSSVILFLNKKDLLEEKIMYSHLISYFPEYTG 293
SP|P30679|GNA15_HUMAN CLEENNQENRMKESLALFGTILELPWFKSTSVILFLNKTDILEEKIPTSHLATYFPSFQG 300
                       * * ::****:** *** **:  *** .:********.*:**:**  ***  ***.: *

SP|P50148|GNAQ_HUMAN  PQRDAQAAREFILKMFVDLNP------------DSDKIIYSHFTCATDTENIRFVFAAVK 345
SP|P29992|GNA11_HUMAN PQRDAQAAREFILKMFVDLNP------------DSDKIIYSHFTCATDTENIRFVFAAVK 345
SP|O95837|GNA14_HUMAN PKQDVRAARDFILKLYQDQNP------------DKEKVIYSHFTCATDTDNIRFVFAAVK 341
SP|P30679|GNA15_HUMAN PKQDAEAAKRFILDMYTRMYTGCVDGPEGSKKGARSRRLFSHYTCATDTQNIRKVFKDVR 360
                      *::*..**: ***.::                   .: ::**:******:*** **  *:

SP|P50148|GNAQ_HUMAN  DTILQLNLKEYNLV 359
SP|P29992|GNA11_HUMAN DTILQLNLKEYNLV 359
SP|O95837|GNA14_HUMAN DTILQLNLREFNLV 355
SP|P30679|GNA15_HUMAN DSVLARYLDEINLL 374
                      *::*   * * **:
```

Fig. S8: Alignment of human Gα_q protein subunits. Amino acid residues within the binding pocket of YM/FR are highlighted in green.

a

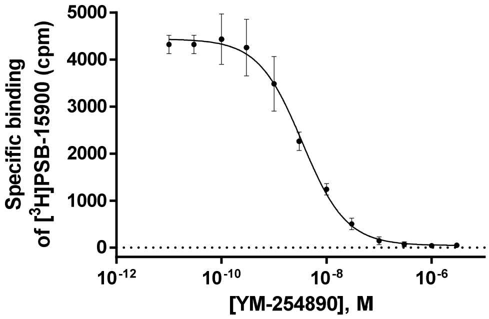

b

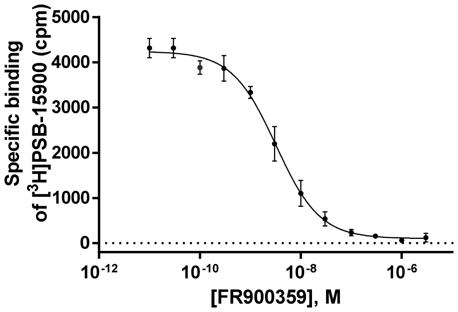

Figure S9: Competition binding studies of YM (a) and FR (b) versus [^{3}H]PSB-15900 (5 nM) in intact human platelets at 37°C. Values represent means ± SD of three experiments. FR exhibited a K_i value of 2.14 ± 0.87 nM (pK_i = 8.68 ± 0.11), YM showed a K_i value of 2.29 ± 0.38 nM (pK_i = 8.68 ± 0.04).

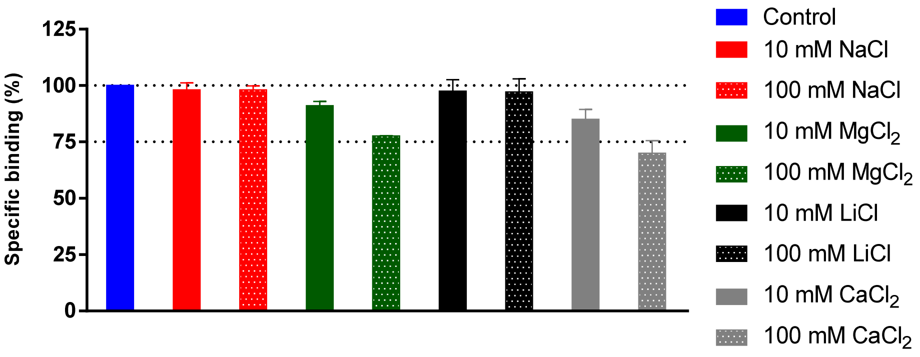

Figure S10: Specific binding of 5 nM [^{3}H]PSB-15900 to human platelet membrane preparations in the presence of selected mono- and divalent metal chlorides. Values represent means ± SD of three independent experiments.

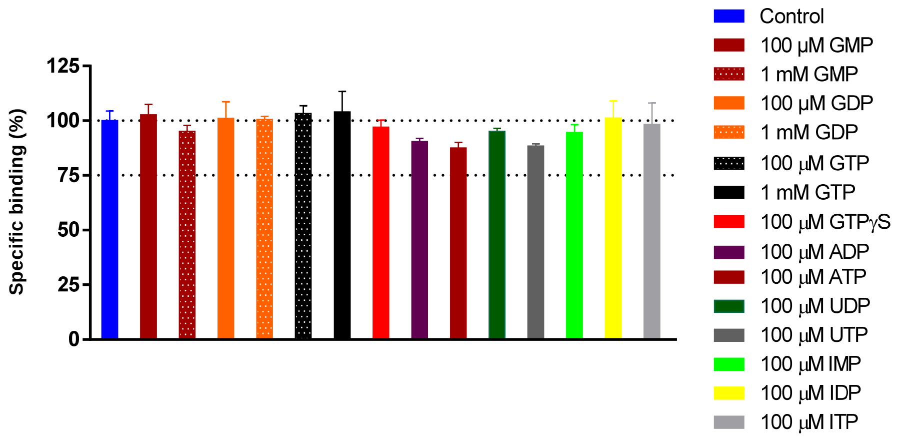

Figure S11: Specific binding of 5 nM [³H]PSB-15900 to human platelet membrane preparations in the presence of nucleotides. Values represent means ± SD of three independent experiments.

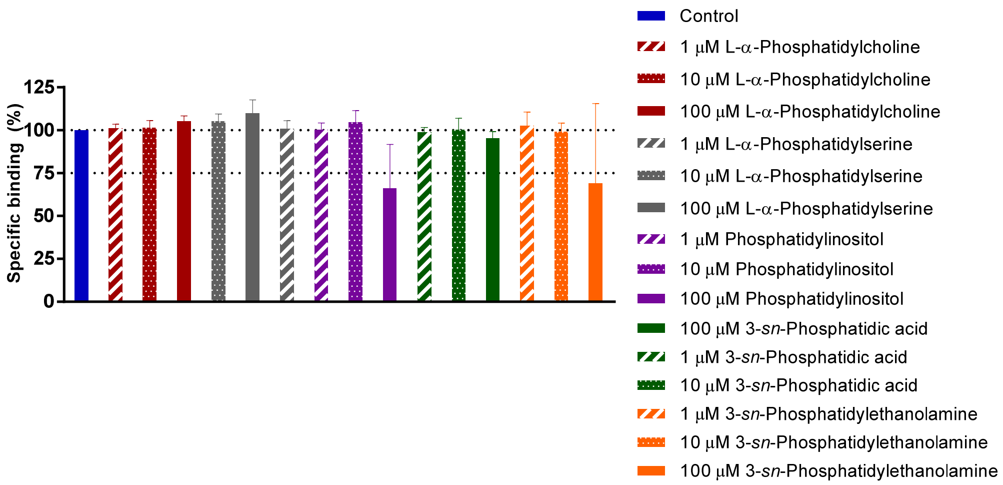

Figure S12: Specific binding of 5 nM [³H]PSB-15900 to human platelet membrane preparations in the presence of phospholipids. Values represent means ± SD of three independent experiments.

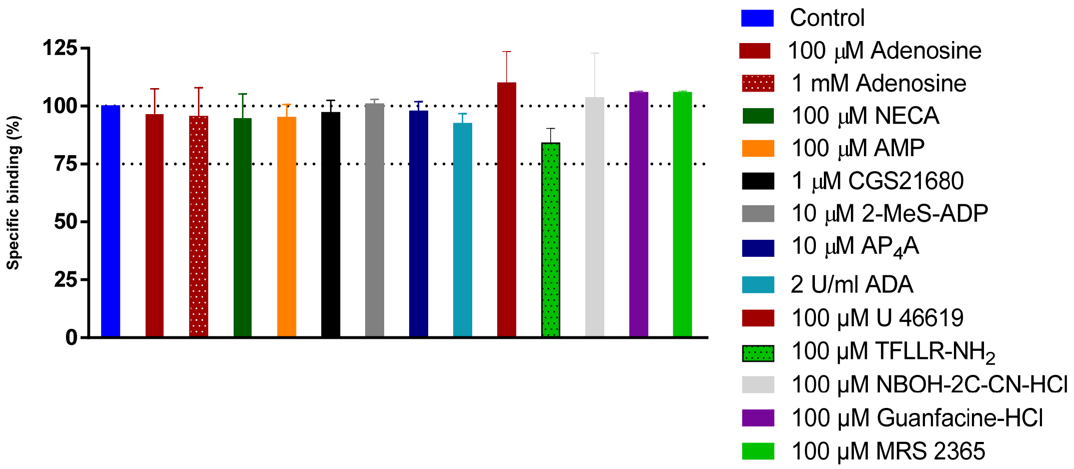

Figure S13: Specific binding of 5 nM [^{3}H]PSB-15900 to human platelet membrane preparations in the presence of GPCR agonists. NECA, N-ethylcarboxamidoadenosine (non-selective adenosine receptor agonist); CGS21680, 3-[4-[2-[[6-amino-9-[(2R,3R,4S,5S)-5-(ethyl-carbamoyl)-3,4-dihydroxyoxolan-2-yl]purin-2-yl]amino]ethyl]phenyl]propanoic acid (A_{2A}-selective adenosine receptor agonist); 2-MeS-ADP, 2-methylthio-ADP ($P2Y_1$ and $P2Y_{12}$ receptor agonist); AP_4A, diadenosine tetraphosphate ($P2Y_2$ receptor agonist); ADA, adenosine deaminase; U 46619, 9,11-dideoxy-9a,11a-methanoepoxyprostaglandin $F_{2\alpha}$ (stable thromboxane A receptor agonist); TFLLR-NH_2, peptide (selective agonist for protease-activated receptor-1 (PAR_1)); NBOH-2C-CN-HCl, 4-[2-[[(2-hydroxyphenyl)methyl]amino]ethyl]-2,5-dimethoxybenzonitrile hydrochloride (selective 5-HT_{2A} receptor agonist); guanfacine-HCl (α_{2A} receptor agonist); MRS 2365, (*N*)-methanocarba-2-methylthio-ADP (selective $P2Y_1$ agonist). Values represent means ± SD of three independent experiments.

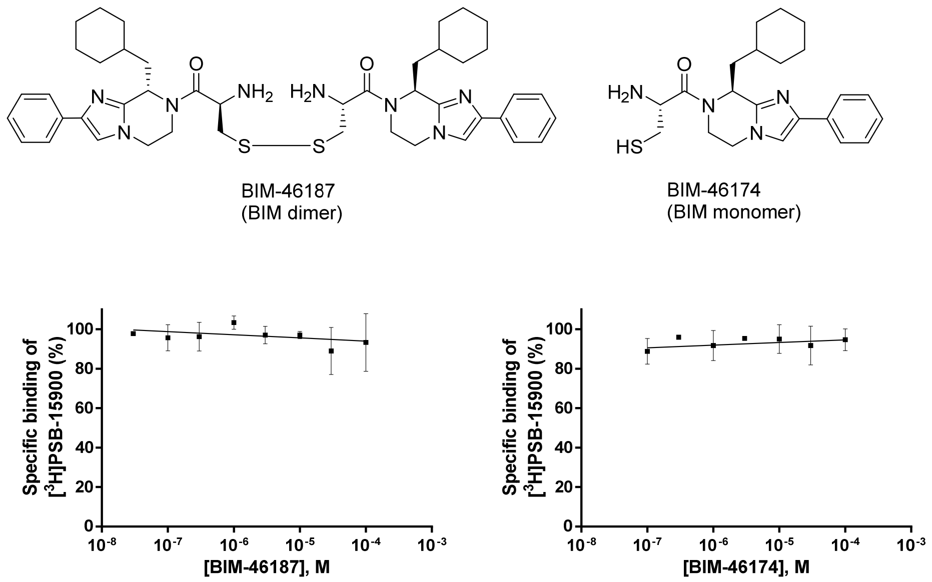

Figure S14: Competition binding studies on intact human platelets with (**A**) BIM-46174 and (**B**) BIM-46187 versus [^{3}H]PSB-15900 (5 nM) at 37°C, preincubation with the G_q inhibitors for 3 h. Values represent means ± SD of three independent experiments.

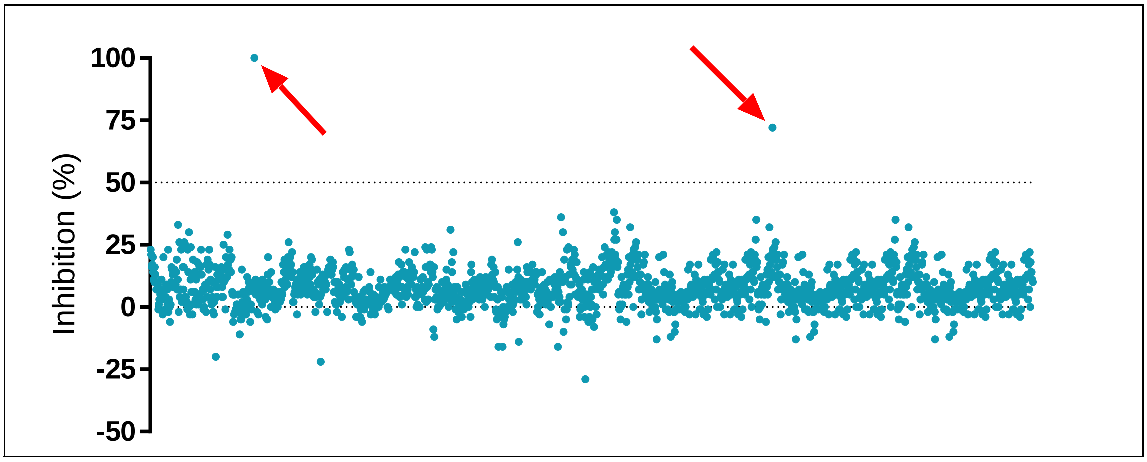

Figure S15: High-throughput screening of compound library. Hits are indicated by a red arrow. Initial screening comprised 2,400 compounds of our in-house compound collection (https://www.pharmchem1.uni-bonn.de/pharmchem1-en/mueller-laboratory/compound-library) that were screened at a concentration of 100 µM in competition binding studies at human platelet membrane preparations (50 µg of protein per vial) versus [^{3}H]PSB-15900 (5 nM). Hits were defined as showing >50% inhibition, which were retested once. For a confirmed hit, concentration-dependent inhibition was determined and the K_i value was calculated. Two hits were identified in the initial screening campaign corresponding to a hit rate of 0.08%.

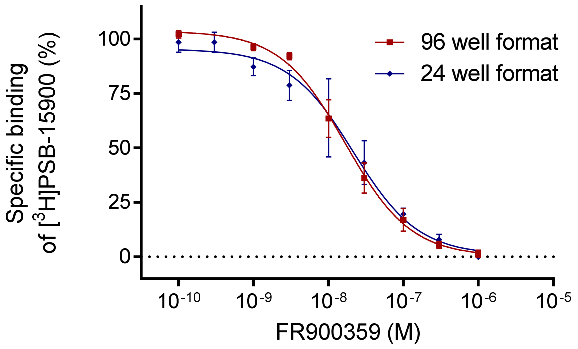

Figure S16: Competition binding studies of FR versus [³H]PSB-15900 (5 nM) performed in a standard 24- and a high-throughput-96-well format at 37°C on human platelet membrane preparations (50 µg of protein/vial). K_i values: 6.91 ± 2.25 nM (pK_i = 8.17 ± 0.15) (24-well format), and 6.56 ± 1.92 nM (pK_i = 8.19 ± 0.13) (96-well format). Values represent means ± SD of three independent experiments.

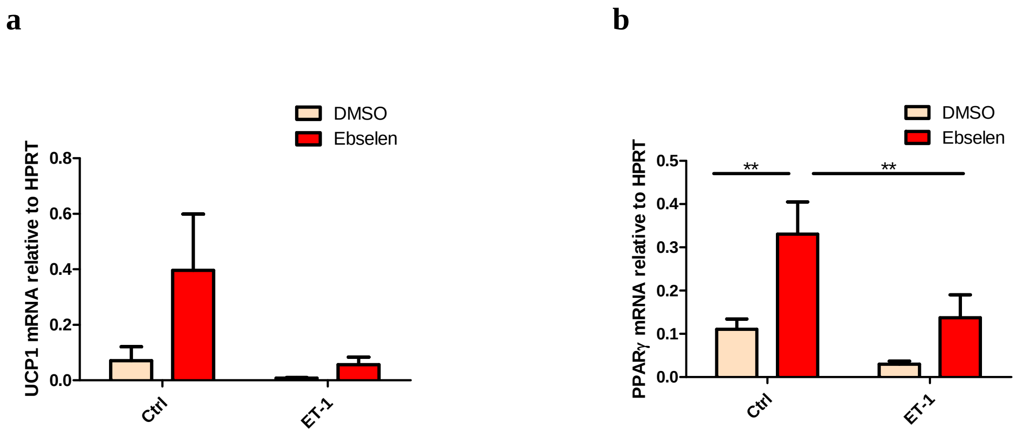

Figure S 17: Brown adipocytes were treated for 9 days with indicated treatments during the differentiation period. mRNA levels of thermogenic marker UCP-1 (**a**) and adipogenic marker PPARγ (**b**) were determined using qPCR. HPRT (hypoxanthine guanine phosphoribosyl

transferase) was used as internal control. Data are shown as means ± SEM from 4 independent experiments, One-way ANOVA with Bonferroni post-hoc test. *$P \leq 0.05$.
